# Supplementary material for: Isotype conversion of Staphylococcal-specific IgG into IgM broadens the reactivity to other bacterial pathogens
Source: Cell Rep Med. 2025 Oct 13;6(10):102414. doi: 10.1016/j.xcrm.2025.102414 (PMC12629822; doi:10.1016/j.xcrm.2025.102414)
Supplement: Document S1. Figures S1–S5 and Tables S1 and S2 [file mmc1.pdf]

Cell Reports Medicine, Volume *6*

Supplemental information

Isotype conversion of Staphylococcal-specific IgG into IgM broadens the reactivity to other bacterial pathogens

Remy M. Muts, Astrid Hendriks, Josefien W. Hommes, Max L.B. Grönloh, Douwe J. Dijkstra, Carla J.C. de Haas, Piet C. Aerts, Eduard H.T.M. Ebberink, Albert J.R. Heck, Zhen Wang, Haoru Zhuang, Jeroen D.C. Codée, Bas G.J. Surewaard, Dani A.C. Heesterbeek, Nina M. van Sorge, and Suzan H.M. Rooijakkers

Supplementary Material

Isotype conversion of Staphylococcal-specific IgG into IgM broadens the reactivity to other bacterial pathogens

Remy M. Muts[1], Astrid Hendriks[2], Josefien W. Hommes[3], Max L.B. Grönloh[1], Douwe J. Dijkstra[1], Carla J.C. de Haas[1], Piet C. Aerts[1], Eduard H.T.M. Ebberink[4], Albert J.R. Heck[4,5], Zhen Wang[6], Haoru Zhuang[6], Jeroen D.C. Codée[6], Bas Surewaard[3], Dani A.C. Heesterbeek[1], Nina M. van Sorge[2,7], Suzan H.M. Rooijakkers[1,*]

[1]*Department of Medical Microbiology, University Medical Center Utrecht, Utrecht, The Netherlands.*
[2]*Department of Medical Microbiology and Infection Prevention, Amsterdam UMC, University of Amsterdam, Amsterdam, The Netherlands.*
[3]*Department of Microbiology, Immunology and Infectious Diseases, Snyder Institute for Chronic Diseases, Cumming School of Medicine, University of Calgary, Calgary, AB T2N 4N1, Canada.*
[4]*Biomolecular Mass Spectrometry and Proteomics, Bijvoet Center for Biomolecular Research and Utrecht Institute of Pharmaceutical Sciences, Utrecht University, 3584 CH Utrecht, The Netherlands.*
[5]*Netherlands Proteomic Center, 3584 CH Utrecht, The Netherlands.*
[6]*Leiden Institute of Chemistry, Leiden University, 2333 CC Leiden, The Netherlands.*
[7]*Netherlands Reference Laboratory for Bacterial Meningitis, Amsterdam UMC, Amsterdam, The Netherlands.*

*Corresponding Author: Suzan Rooijakkers, Email: s.h.m.rooijakkers@umcutrecht.nl

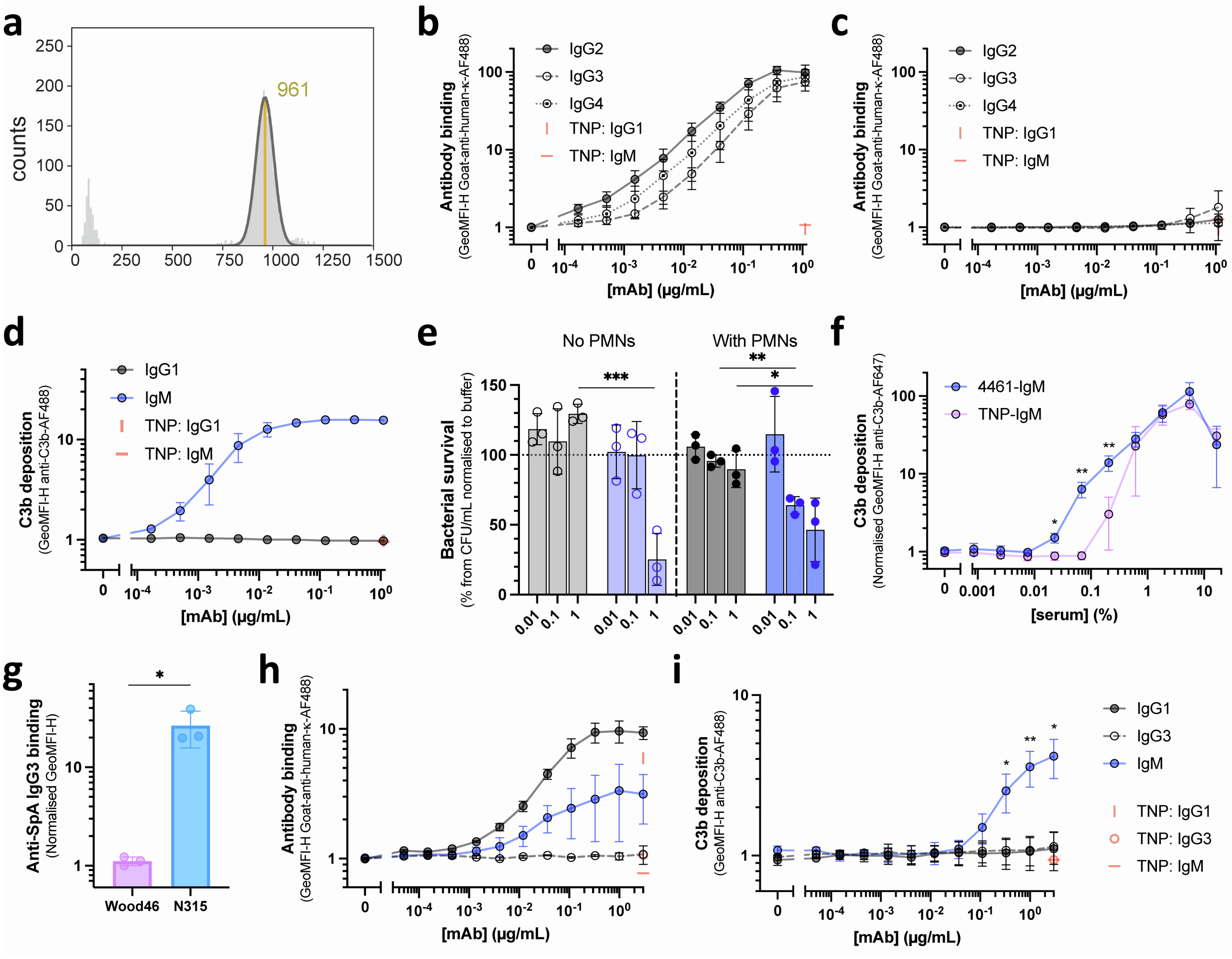

Figure S1. Control experiments to validate the quality, specificity, and functionality of the recombinant IgMs. Related to figure 1.

a) Mass photometry analysis of recombinant pentameric IgM. Mass histograms with Gaussian fits of the peaks of mass photometry analysis of recombinant anti-WTA (4461) IgM co-expressed in the presence of a J-chain. Bandwiths of 10 kDa were used and annotated mass correspond to local maxima in density. A minor peak was observed below 90 kDa, this is most likely not a real binding event, but generated as artefacts of background interactions with the glass plate and are typically observed on the Refeyn MP instrument used here. **b, c) 4461 IgG2-4 binding to *S. aureus* LAC and Wood46.** Concentration-dependent binding of anti-WTA (4461) IgG2, IgG3, and IgG4 and 1 µg/mL isotype controls anti-TNP IgG1 and IgM to b) *S. aureus* LAC Δspa, sbi::Tn or to c) *S. aureus* Wood46. **d, e) 4461 IgG1 and IgM mediated complement activation and OPK.** d) C3b deposition induced by a concentration range of anti-WTA (4461) IgG1 and IgM in 1 % ΔIgG/M-serum on Wood46. As isotype controls, 1 µg/mL anti-TNP IgG1 and IgM were included. e) Normalised CFU/mL values over buffer control of *S. aureus* Wood46 incubated in a opsonophagocytic killing (OPK) killing assay with human neutrophils. Bacteria were first incubated with the in the graph indicated concentration in µg/mL of either 4461-IgG1 (in grey) or 4461-IgM (in blue) with 1% ΔIgG/M-serum or buffer, and subsequently either with or without human PMNs. After incubation the neutrophils were lysed with saponin and remaining bacteria were plated and grown overnight before CFU enumaration. A multiple unpaired t test was used to determine significant differences in which p-values are indicated with * < 0.05, ** < 0.01, or *** < 0.001. **f) 4461-IgM can induce complement activation in the presence of competing antibodies.** C3b deposition induced by 1 µg/mL anti-WTA (4461) IgM or anti-TNP IgM in a concentration range of health donor serum on *S. aureus* Wood46. A multiple unpaired t test was used to determine significant differences between the antibodies at erach concentration in which p-values are indicated with * < 0.05, or ** < 0.01. **g – i) 4461-IgM cross-reactive binding and complement activation occur despite the expression of SpA.** g) Detection of SpA expression with an anti-SpA IgG3 antibody of *S. aureus* Wood46 or N315. Values were normalised over an anti-DNP IgG3 antibody control. A multiple unpaired t test was used to determine significant differences between the strain in which p-value is indicated with * < 0.05. h) Concentration-dependent binding of anti-WTA (4461) IgG1, IgG3, and IgM and anti-TNP isotype controls to *S. aureus* N315. i) C3b deposition induced by a concentration range of anti-WTA (4461) IgG1, IgG3, and IgM and anti-TNP isotype controls 1 % ΔIgG/M-serum on N315. A multiple unpaired t test was used to determine significant differences between IgG1 and IgM at each concentration in which p-values are indicated with * < 0.05, or ** < 0.01. All data represents mean ± SD of three independent experiments.

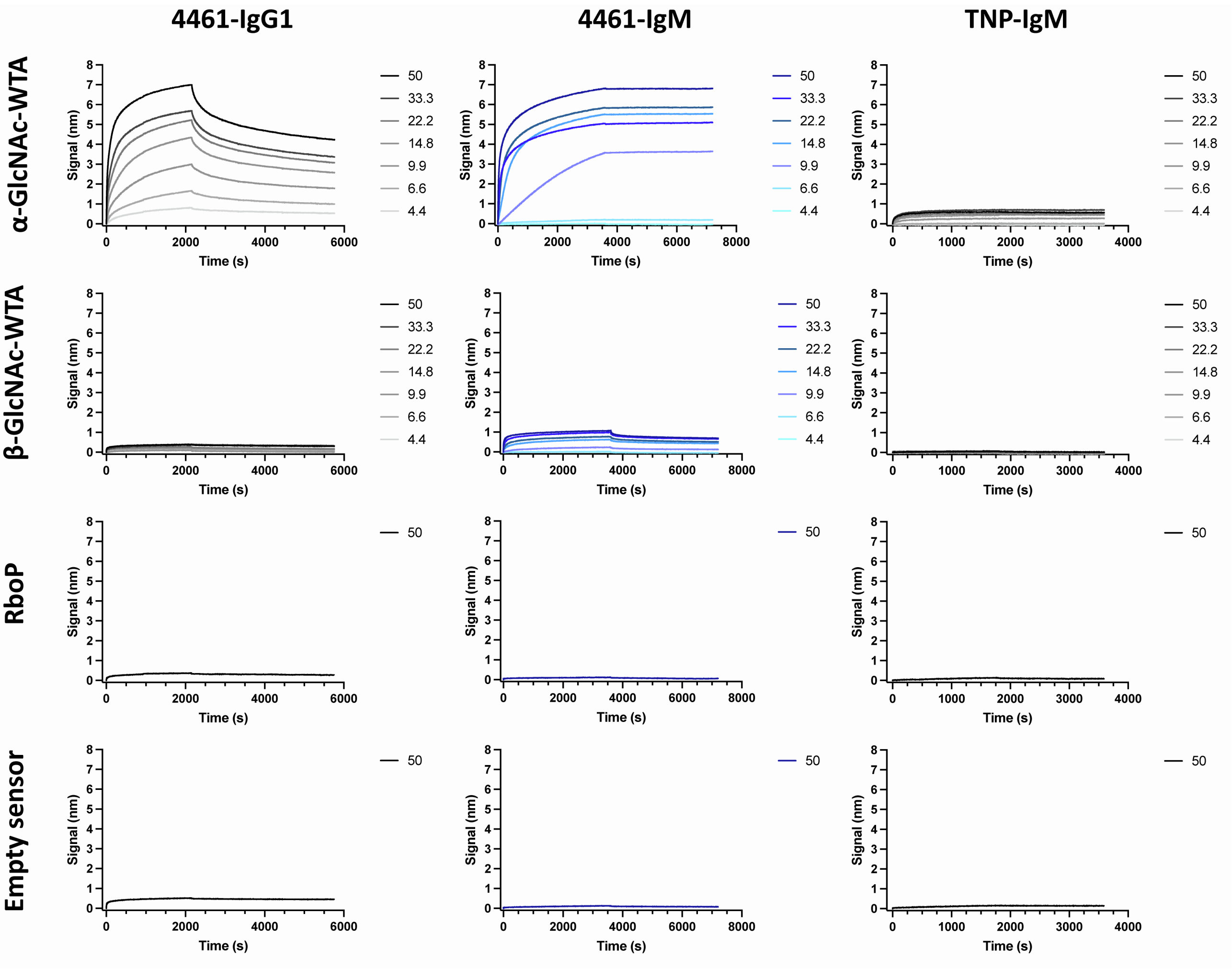

Figure S2. Bio-layer interferometry confirms 4461-IgM cross-reactivity. Related to figure 1.
Bio-layer interferometry measurement of different concentrations of 4461-IgG1, 4461-IgM, and TNP-IgM to streptavidin sensors loaded with either 1 μM biotinylated α-GlcNAc-WTA hexamers, β-GlcNAc-WTA hexamers, RboP hexamers, or nothing. 4461-IgG1 association was measured for 2150 sec, and dissociation for 3600 sec. 4461-IgM association and dissociation were measured for 3600 sec. TNP-IgM association and dissociation were measured for 1800 sec. Legends indicate antibody concentrations in μg/mL.

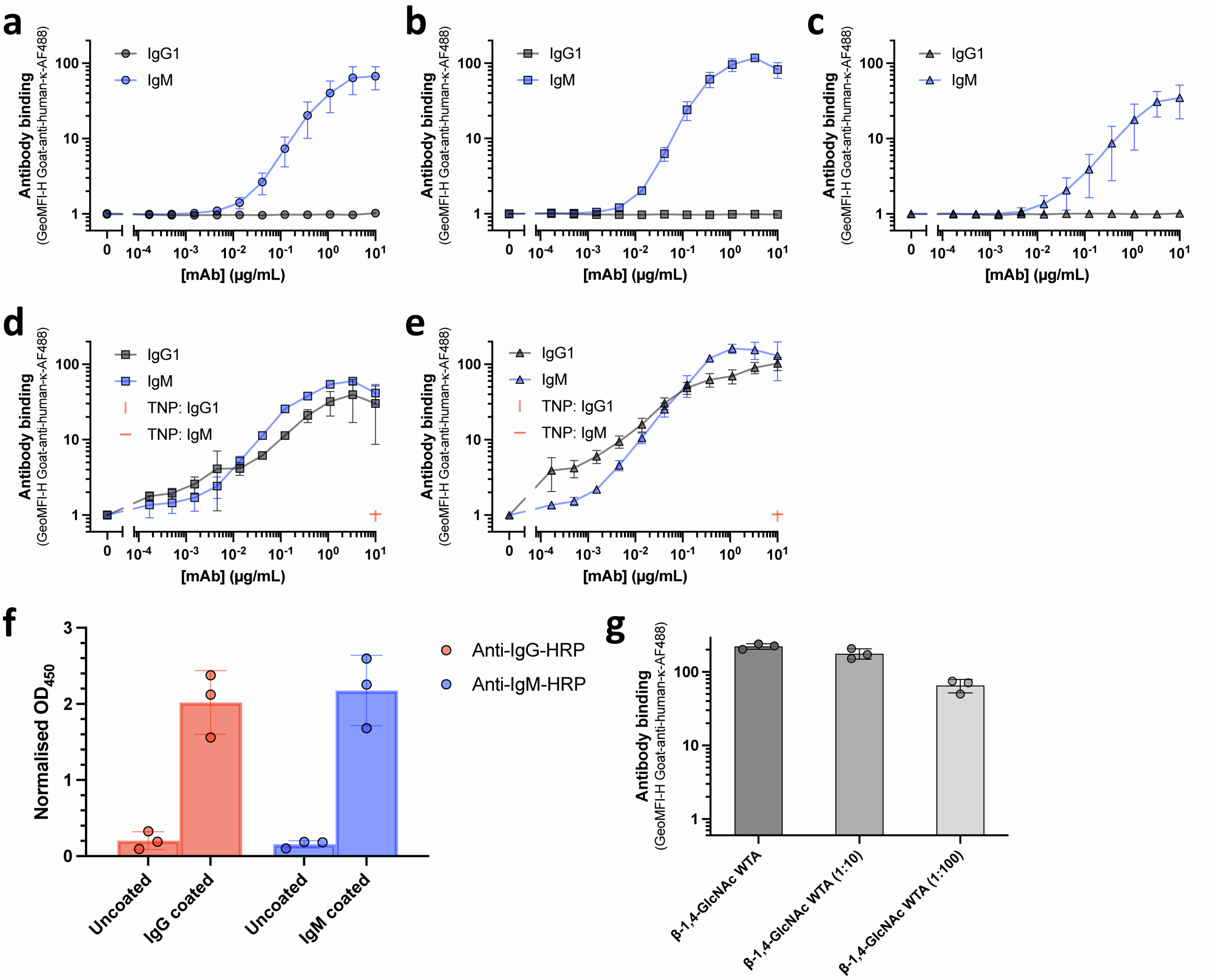

Figure S3. Dose-response curves and controls of antibody binding to *E. coli*, *S. aureus*, and WTA beads. Related to figure 2 and 3.

a – c) 4461, 4997, and rF1 IgG1 and IgM binding curves to *E. coli* MG1655. Concentration-dependent binding of a) anti-WTA (4461), b) anti-WTA (4497), and c) anti-SDR (rF1) IgG1 and IgM to *E. coli* MG1655. **d, e) Validation of 4497 and rF1 IgG1 and IgM binding to *S. aureus*.** Concentration-dependent binding of d) anti-WTA (4497) and e) anti-SDR (rF1) IgG1 and IgM to *S. aureus* LAC Δspa, sbi::Tn. **f) Controls for the serum IgG- and IgM-coated ELISA.** Controls for ELISA with coated serum IgG and IgM. To verify that the coating was successful, the binding of Goat-anti-human-IgG-HRP was assessed to uncoated wells or wells coated with 3 μg/mL serum IgG. As functional control for the anti-IgM detection antibody, its binding was assessed to either uncoated wells, or wells coated with 3 μg/mL serum IgM. **g) 4497-IgG1 binding to horizontaly antigen reduced synthetic WTA beads remains.** Binding of 1 μg/mL anti-WTA (4497) to WTA beads coated with a 10-fold and 100-fold dilution (compared to standard) of synthetic RboP hexamers with one terminal β-1,4-GlcNAc modification. All data represents mean ± SD of three independent experiments.

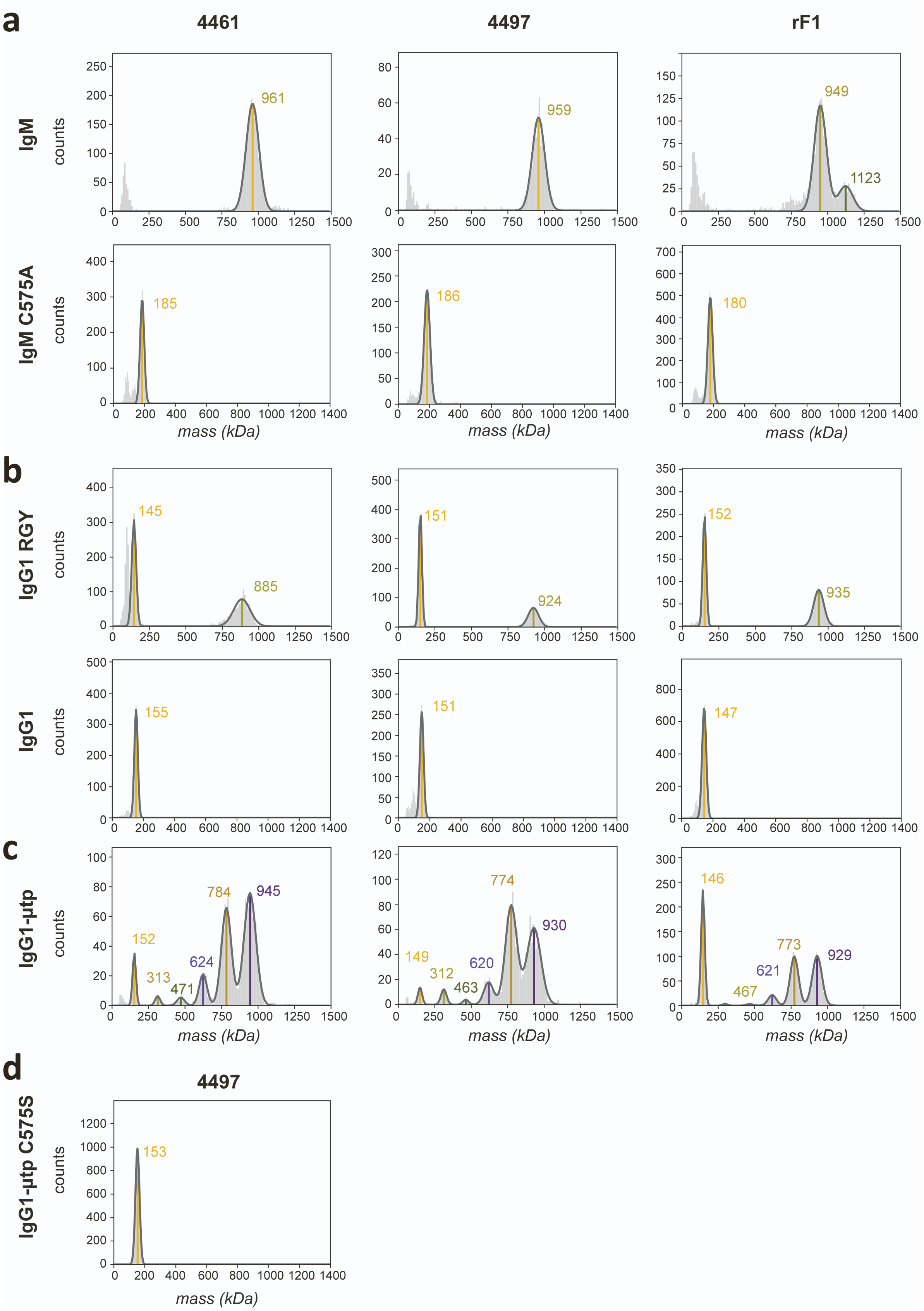

Figure S4. Mass photometry analysis of Ig-constructs to verify oligomerisation status. Related to figure 4.
Mass histograms with Gaussian fits of the peaks of mass photometry analysis of recombinant: a) 4461, 4497, and rF1 IgM co-expressed in the presence of a J-chain and IgM C575A monomers; b) IgG1 RGY and IgG1; c) IgG1-µtp; and d) 4497 IgG1-µtp C575S. 4461 IgM is also presented in supplemental figure 1 and is shown here as comparison to 4461 IgM C575A. Bandwiths of 10 kDa were used for IgMs, IgG1-µtps, and IgG1-RGYs or 5 kDA for IgG1s, IgM C575A monomers, and IgG1-µtp C575S. Annotated masses correspond to local maxima in density. In some of the mass histograms minor peaks were observed below 90 kDa. These are most likely not real binding events, but generated as artefacts of background interactions with the glass plate and are typically observed on the Refeyn MP instrument used.

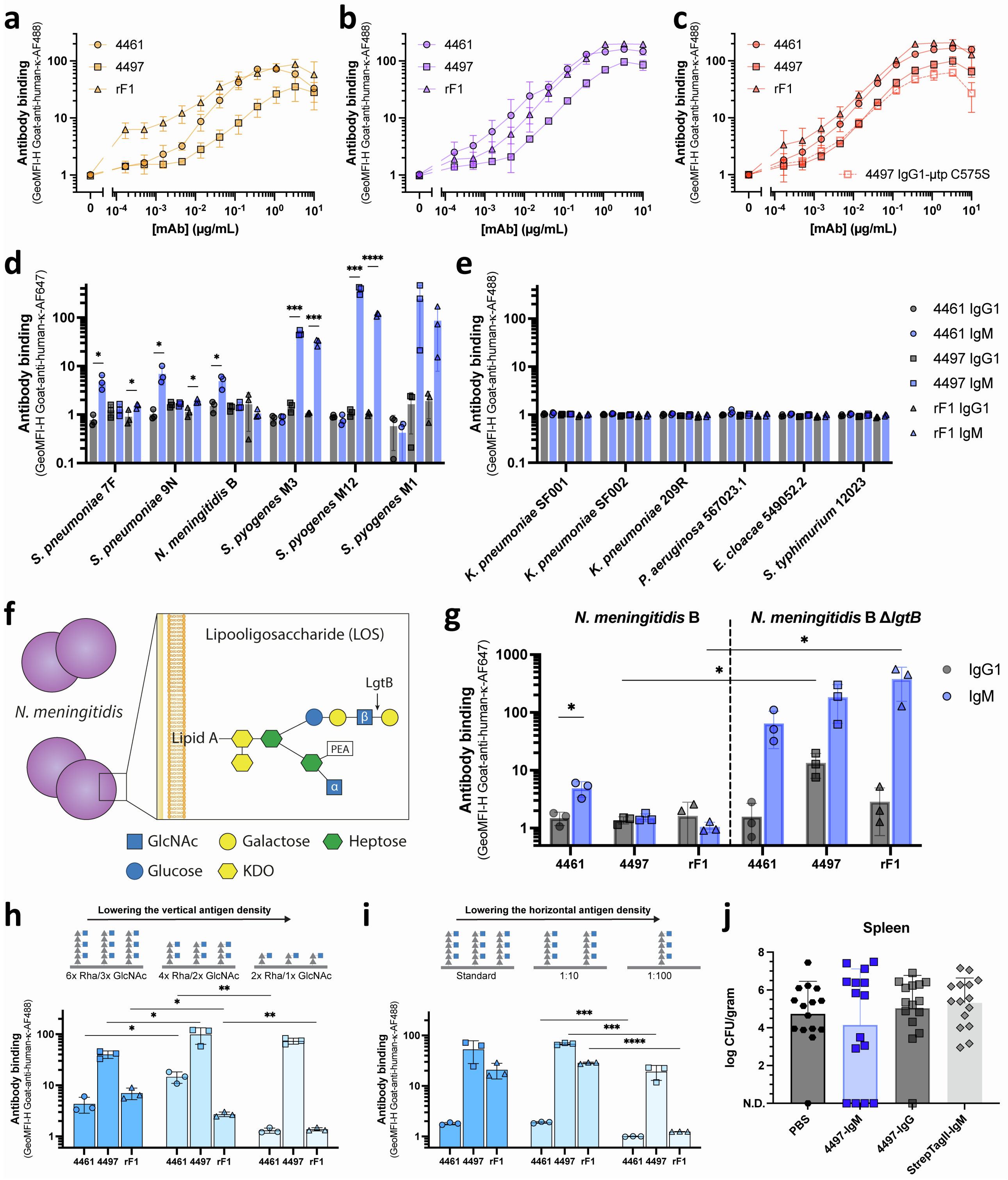

Figure S5. Control experiments related to figure 4 and 5.
a – c) IgG engineered mAbs can bind their original target on *S. aureus*. Binding of 4461, 4497, and rF1 across a concentration range and engineered with the following mutations a) IgM C575A, or b) IgG1 RGY, or c) IgG1-µtp and 4497 IgG1-µtp C575S to *S. aureus* LACΔSpA, sbi::Tn. **d, e) Converted IgMs cross-react to species with surface-expressed GlcNAc.** Binding of 1 µg/mL anti-WTA (4461), anti-WTA (4497), and anti-SDR (rF1) IgG1 and IgM to a range of bacterial species with d) described surface-expressed GlcNAc or e) clinical isolates without described GlcNAc moiety on their surface. A multiple unpaired t test was used to determine significant difference between IgG1 and IgM for each mAb per strain in which p-values are indicated with * < 0.05, ** < 0.01, *** < 0.001 and **** < 0.0001. **f, g) IgM cross-reactivity to *N. meningitidis* is partially obscured by a galactose moiety.** f) Schematic representation of the structure and glycosylation of *N. meningitidis* serogroup B LOS and the incorporation of galactose by LgtB on top of the β-GlcNAc is indicated with an arrow. PEA: phosphoethanolamine. g) Binding of 1 µg/mL 4461, 4497, and rF1 IgG1 and IgM to *N. meningitidis* B Wt (left) and ΔlgtB (right). A multiple unpaired t test was used to determine significant difference between the indicated bar graphs in which p-values are indicated with * < 0.05. **h, i) A high antigen density**

is required for IgM cross-reactivity to GAC beads. h) Binding of anti-WTA (4461), anti-WTA (4497), and anti-SDR (rF1) IgM (1 µg/mL) to GAC beads coated with completely defined synthetic: rhamnose (Rha) hexamers with three β-1,3-GlcNAc modifications, rhamnose tetramers with two GlcNAcs, or rhamnose dimers with one GlcNAc. i) Binding of anti-WTA (4461), anti-WTA (4497), and anti-SDR (rF1) IgM (1 µg/mL) to GAC beads coated with a 10 or 100-fold dilution compared to the standard of completely defined synthetic Rha hexamers with three β-1,3-GlcNAc modifications. h) and i) contain a schematic representation of the variation in the vertical and horizontal antigen density on GAC beads. Gray triangles represent Rha monomers, and blue squares GlcNAc moieties. A multiple unpaired t test was used to determine significant difference between the antigen densities in which p-values are indicated with * < 0.05, ** < 0.01, *** < 0.001 and **** < 0.0001. All data represents mean ± SD of three independent experiments**. j) Spleen CFU of mice infected with *S. pyogenes*.** Colony forming units (CFU) in spleen of mice (n = 15 per group) 24 h post infection with *S. pyogenes* 5448 (±5*10^7 CFU), passively immunised with 50 µg 4497-IgM, 4497-IgG1, anti-StrepTagII-IgM, or PBS.

Table S1. Related to STAR Methods. Bacterial species and strains used.

Bacterial strain	Plate type	Growth medium	Antibiotic	Source	OD_{600} count
S. aureus LAC Δ*spa sbi*::Tn	Blood agar	Todd Hewitt Broth		[61]	
S. aureus Wood46 cru006	Blood agar	Todd Hewitt Broth		[61]	6E8 / mL
S. aureus Wood46 cru006 GFP	Blood agar	Todd Hewitt Broth	10 µg/mL Chloramphenicol	[61]	
E. coli MG1655	LB agar	Lysogeny Broth		[40]	4E8 / mL
E. coli BW25113 Wt	LB agar	Lysogeny Broth		Keio library	
E. coli BW25113 Δ*WaaR*	LB agar	Lysogeny Broth	10 µg/mL Kanamycin	Keio library	
E. coli BW25113 Δ*WaaB*	LB agar	Lysogeny Broth	10 µg/mL Kanamycin	Keio library	
E. coli CGSC7740 Wt (*wbbL*-)	LB agar	Lysogeny Broth		^	
E. coli CGSC7740 *wbbL*+	LB agar	Lysogeny Broth		^	
S. pneumoniae 7F	Blood agar	THY* 5% CO_2		NRLBM**	2E8 / mL
S. pneumoniae 9N	Blood agar	THY* 5% CO_2		NRLBM**	
N. meningitidis B H44/76	Blood agar	Tryptic Soy Broth		NRLBM**	2E8 / mL
N. meningitidis B H44/76 Δ*lgtB*	Blood agar	Tryptic Soy Broth	100 µg/mL Kanamycin	^^	
S. pyogenes M3	Blood agar	THY* 5% CO_2		^^^	
S. pyogenes M12	Blood agar	THY* 5% CO_2		NRLBM**	
S. pyogenes M1 5448	Blood agar	THY* 5% CO_2		[62]	5E7 / mL
K. pneumoniae 209S	LB agar	Lysogeny Broth		UMC Utrecht	3E8 / mL
K. pneumoniae SF001	LB agar	Lysogeny Broth		UMC Utrecht	
K. pneumoniae SF002	LB agar	Lysogeny Broth		UMC Utrecht	
P. aeruginosa 567023.1	LB agar	Lysogeny Broth		UMC Utrecht	1E8 / mL
E. cloacae 549052.2	Blood agar	Tryptic Soy Broth		UMC Utrecht	2E8 / mL
S. typhimurium 12023	LB agar	Tryptic Soy Broth		UMC Utrecht	1E8 / mL

^ Kindly provided by Benjamin Sellner, Biozentrum, University of Basel.
^^ Kindly provided by Peter van der Ley, Intravacc.
^^^ Kindly provided by Gunnar Lindahl, Lund University.
* THY = Todd Hewitt Broth with 0.5% Yeast extract
** Netherlands Reference Laboratory for Bacterial Meningitis

Table S2. Related to STAR Methods. Variable heavy (VH) and variable light (VL) chain region amino acid sequences for each antibody.

Antibody	VH	VL	Source
Anti-WTA (4461)	QVQLVQSGAEVRKPGASVKVSCKASGYSFTDYYMHWVRQAPGQGLEWMGWINPKSGGTNYAQRFQGRVTMTGDTSISAAYMDLASLTSDDTAVYYCVKDCGSGGLRDFWGQGTTVTVSS	DIQMTQSPDSLAVSLGERATINCKSSQSVLSRANNNYYVAWYQHKPGQPPKLLIYWASTREFGVPDRFSGSGSGTDFTLTINSLQAEDVAVYYCQQYYTSRRTFGQGTKVEIK	[8]
Anti-WTA (4497)	EVQLVESGGGLVQPGGSLRLSCSASGFSFNSFWMHWVRQVPGKGLVWISFTNNEGTTTAYADSVRGRFIISRDNAKNTLYLEMNNLRGEDTAVYYCARGDGGLDDWGQGTLVTVSS	DIQLTQSPDSLAVSLGERATINCKSSQSIFRTSRNKNLLNWYQQRPGQPPRLLIHWASTRKSGVPDRFSGSGFGTDFTLTITSLQAEDVAIYYCQQYFSPPYTFGQGTKLEIK	[28]
Anti-SDR (rF1)	EVQLVESGGGLVQPGGSLRLSCAASGFTLSRFAMSWVRQAPGRGLEWVASINSGNNPYYARSVQYRFTVSRDVSQNTVSLQMNNLRAEDSATYFCAKDHPSSGWPTFDSW GPGTLVTVSS	DIQLTQSPSALPASVGDRVSITCRASENVGDWLAWYRQKPGKAPNLLIYKTSILESGVPSRFSGSGSGTEFTLTISSLQPDDFATYYCQHYMRFPYTFGQGTKVEIK	[36]
Anti-StrepTagII	EVQLEQSGPELVKPGASVKMSCKASGYTFTNYYMKWVKQSHGKSLEWIGDLNPNNGDTFYNQKFKGKATLTVDKSSNTAYMQLNSLTSEDSAVYYCARTGRYEENAMDYWGQGTSVTVSS	ELVMTQSPASLAVSLGQRATISCRASESVDSYGKSFMHWYQLKPGQPPKLLIYRASNLESGVPARFSGSGSRTDFTLTIDPVEADDAATYYCQQNNEDPWTFGGGTKLEIK	WO 2015/067768A1
Anti-TNP	EVQIQESGPSLVKPSQTLSLTCSVSGDSITSGYWNWIRKFPGHKIEYMGTISYSGDTYYNPSLKSRISITRDTSKNQYYLHLNSVTTEDTATYYCARYGSYVFDYWGQGTTLTVSS	DVVMTQTPLSLPVSLGDQASISCRSSQSLLHSNGNTYLHWYLQKPGQSPKLLIYKVSNRFSGVPDRFSGSGSGTDFTLKISRVEAEDLGVYFCSQSTHVPWTFGGGTKLEIK	*
Anti-ClfA (Aurex)	QVQLKESGPGLVAPSQSLSITCAISGFSLSRYSVHWVRQPPGKGLEWLGMIWGGGNTDYNSALKSRLSISKDNSKSQVFLKMNSLQTDDTAMYYCARKGEFYYGYDGFVYWGQGTLVTVSA	NIMMTQSPSSLAVSAGEKVTMSCKSSQSVLYSSNQKNYLAWYQQKPGQSPKLLIYWASTRESGVPDRFTGSGSGTDFTLTISSVQAEDLAVYYCHQYLSSYTFGGGTKLEIK	WO2002/WO02072600A2
Anti-CD52 (Campath)	QVQLQESGPGLVRPSQTLSLTCTVSGFTFTDFYMNWVRQPPGRGLEWIGFIRDKAKGYTTEYNPSVKGRVTMLVDTSKNQFSLRLSSVTAADTAVYYCAREGHTAAPFDYWGQGSLVTVSS	DIQMTQSPSSLSASVGDRVTITCKASQNIDKYLNWYQQKPGKAPKLLIYNTNNLQTGVPSRFSGSGSGTDFTFTISSLQPEDIATYYCLQHISRPRTFGQGTKVEIK	WO 2013/181568A2
Anti-SpA (10919)	EVQLVQSGAEVKKPGASVKVSCKASGYTFTSYYMHWVRQAPGQGLEWMGIINPRVGSTSYAQKFQGRVTMTRDTSTSTVYMELSSLRSEDTAVYYCARGRPLSGTGGHHYFDYWGQGTLVTVSS	EIVLTQSPATLSVSPGERATLSCQASQDISNYLNWYQQKPGQAPRLLIYDASNLETGIPARFSGSGSGTEFTLTISSLQSEDFAVYYCQQVYALPPWTFGGGTKVEIK	US 2018/0105584A1

*Kindly provided by Gestur Vidarsson, Sanquin.
