# Supplementary material for: Isotype conversion of Staphylococcal-specific IgG into IgM broadens the reactivity to other bacterial pathogens
Source: Cell Rep Med. 2025 Oct 13;6(10):102414. doi: 10.1016/j.xcrm.2025.102414 (PMC12629822; doi:10.1016/j.xcrm.2025.102414)
Supplement: Document S2. Article plus supplemental information [file mmc2.pdf]

Cell Reports Medicine

Article

Isotype conversion of Staphylococcal-specific IgG into IgM broadens the reactivity to other bacterial pathogens

Graphical abstract

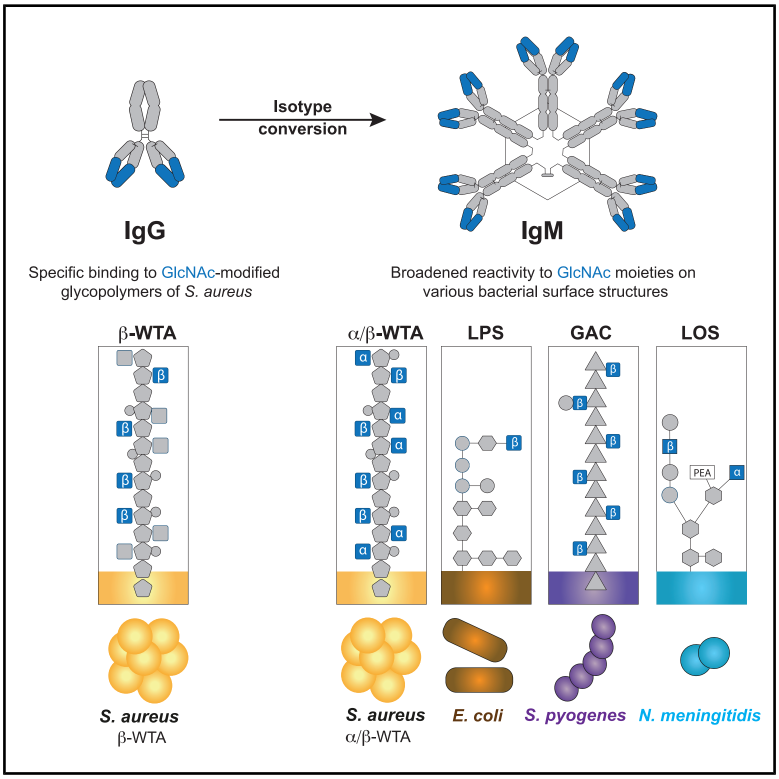

Authors

Remy M. Muts, Astrid Hendriks, Josefien W. Hommes, ..., Dani A. C. Heesterbeek, Nina M. van Sorge, Suzan H.M. Rooijakkers

Correspondence

s.h.m.rooijakkers@umcutrecht.nl

In brief

The narrow antigen specificity of IgGs hampers their utility for antibacterial therapies. Muts et al. show that conversion of highly specific IgGs into IgM induces broader reactivity toward several human pathogens while preserving functional activity. These findings may accelerate development of broadly reactive antibodies to prevent and treat bacterial infections.

Highlights

- IgGs considered specific for *S. aureus* cross-react to other human pathogens as IgM
- Cross-reactive IgMs recognize several human pathogens with exposed GlcNAc moieties
- These IgMs induce complement effects on Gram-positive and Gram-negative bacteria
- IgM cross-reactivity requires multivalency and can be mimicked by IgG engineering

Muts et al., 2025, Cell Reports Medicine *6*, 102414
October 21, 2025 © 2025 The Authors. Published by Elsevier Inc.
https://doi.org/10.1016/j.xcrm.2025.102414

CellPress

Cell Reports Medicine

CellPress
OPEN ACCESS

Article

Isotype conversion of Staphylococcal-specific IgG into IgM broadens the reactivity to other bacterial pathogens

Remy M. Muts,[1] Astrid Hendriks,[2] Josefien W. Hommes,[3] Max L.B. Grönloh,[1] Douwe J. Dijkstra,[1] Carla J.C. de Haas,[1] Piet C. Aerts,[1] Eduard H.T.M. Ebberink,[4] Albert J.R. Heck,[4,5] Zhen Wang,[6] Haoru Zhuang,[6] Jeroen D.C. Codée,[6] Bas G.J. Surewaard,[3] Dani A.C. Heesterbeek,[1] Nina M. van Sorge,[2,7] and Suzan H.M. Rooijakkers[1,8,*]

[1]Department of Medical Microbiology, University Medical Center Utrecht, Utrecht, the Netherlands
[2]Department of Medical Microbiology and Infection Prevention, Amsterdam UMC, University of Amsterdam, Amsterdam, the Netherlands
[3]Department of Microbiology, Immunology and Infectious Diseases, Snyder Institute for Chronic Diseases, Cumming School of Medicine, University of Calgary, Calgary, AB T2N 4N1, Canada
[4]Biomolecular Mass Spectrometry and Proteomics, Bijvoet Center for Biomolecular Research and Utrecht Institute of Pharmaceutical Sciences, Utrecht University, 3584 CH Utrecht, the Netherlands
[5]Netherlands Proteomic Center, 3584 CH Utrecht, the Netherlands
[6]Leiden Institute of Chemistry, Leiden University, 2333 CC Leiden, the Netherlands
[7]Netherlands Reference Laboratory for Bacterial Meningitis, Amsterdam UMC, Amsterdam, the Netherlands
[8]Lead contact
*Correspondence: s.h.m.rooijakkers@umcutrecht.nl
https://doi.org/10.1016/j.xcrm.2025.102414

SUMMARY

Therapeutic antibodies are actively explored as alternative to treat or prevent bacterial infections. However, the narrow antigen specificity of IgG in combination with broad diversity in bacterial surface structures currently hampers the development of therapeutic antibodies against bacteria. Here we reveal that isotype conversion of three highly specific anti-staphylococcal antibodies from IgG into IgM does not only affect Fc effector functions but also modifies the interaction of Fab domains with bacterial surface antigens. These converted IgMs gain cross-reactivity for a broad range of bacterial species, including Gram-negatives such as *Escherichia coli* and *Neisseria meningitidis* and even protect against invasive infection with *Streptococcus pyogenes in vivo*. Mechanistic studies show that enhanced cross-specificity by IgM is conferred by changed ligand specificity and multivalent binding to high-density antigens. Altogether, these findings provide important insights for the development of antibody therapy for bacterial infections.

INTRODUCTION

Due to the current rise in antibiotic resistance among bacteria,[1] therapeutic antibodies are actively explored as an alternative approach to treat or prevent bacterial infections.[2,3] Antibodies, or immunoglobulins (Ig), are a vital part of host protection against infections by connecting recognition of foreign antigens via their variable (Fab) domains to cellular and humoral immune effector functions through their constant (Fc) region.[4] In the past years, the discovery of monoclonal antibodies (mAbs) was boosted by the advancement of methods to sequence antigen-binding domains from single cell-sorted B cells of previously exposed human individuals.[5] This technological advance in combination with the incredible success of antibody therapies in treatment of specific cancers and autoimmune diseases[6,7] has sparked interest of their therapeutic application in infectious diseases.[2,3]

Despite a clear medical need,[1] the development of antibody therapies for bacterial infections is still lagging behind other fields. In the past 5–10 years, a considerable number of anti-bacterial mAbs has been discovered, especially against the highly virulent and antibiotic-resistant pathogens of the ESKAPE group.[8–11] Next to toxin-neutralizing antibodies, there is a strong interest in the development of immune-activating antibodies since these can engage Fc-mediated effector functions that are critical to kill bacteria.[9,10,12–15] To do this, antibodies should first bind to the bacterial surface via their antigen-specific Fab domain. Next, the antibody's Fc domain can engage Fcγ-receptors directly or activate complement,[4,16] which potently stimulates phagocytosis and intracellular killing.[14,16] Also, complement directly kills Gram-negative bacteria via formation of membrane attack complex (MAC) pores.[17] As for many other diseases, most pharmaceutical and academic antibody programs in infectious diseases focus on discovering IgG antibodies. IgGs are considered superior for clinical development due to their higher specificity (and affinity) for their antigen through affinity selection and somatic hypermutations in combination with the ample available know-how on large-scale clinical grade production.[4,5,7]

The high antigen specificity of IgG is generally an advantage (e.g., in the case of cancer). However, it poses a limitation for

© 2025 The Authors. Published by Elsevier Inc.
This is an open access article under the CC BY license (http://creativecommons.org/licenses/by/4.0/).

the development of effective antibodies against bacteria, since bacterial pathogens can express highly variable surface structures, even when belonging to the same species. Consequently, monoclonal IgGs targeting a bacterial surface structure may not cover the strain diversity within a single species. For example, *Klebsiella pneumoniae* expresses over 77 different capsule types and 8 different O-antigen types.[18] Monoclonals targeting a certain capsule type or O-type, will not cross-bind to other *K. pneumoniae* types.[9,10] Similarly, *Streptococcus pneumoniae* expresses almost 100 different capsule types and IgGs against a specific capsule type rarely react with other variants.[19,20] In summary, the narrow antigen specificity of IgGs in combination with high diversity in bacterial surface structures currently hampers the development of therapeutic antibodies against bacteria.

To overcome the current limitations of IgG, we considered other Ig isotypes such as IgM, which is largely understudied in immunology and infectious diseases.[21,22] IgM, the first antibody isotype to be produced during a natural infection, circulates as a preassembled oligomer of five IgM monomers (pentamer).[23] Although single IgM-Fab domains have a lower affinity for their antigen than IgG-Fabs, its pentameric structure allows multivalent interaction with high density antigens,[21,22] resulting in high avidity interactions. IgM's low affinity and shorter half-life (compared to IgG) have so far tempered enthusiasm for the development of IgM as a therapeutic agent.[21,22] Nevertheless, it is long known that IgM is crucial for natural immune protection against bacterial infections.[22] Also, more recent studies showed that IgM is critical for human immunity against *S. aureus* and provides a higher correlate of protection after vaccination against *S. pneumoniae*.[24,25] Additionally, interest in IgM was recently boosted because IgM showed a broader protective capacity against different SARS-CoV-2 variants than IgG.[26] These findings suggest that exploring the mechanisms by which IgM mediates bacterial protection could be important for future development of antibody therapies against bacteria.

Here, we uncovered that *in vitro* conversion of highly specific anti-bacterial IgGs into IgM did not only affect Fc effector functions, but also changed the interaction of Fab domains with the bacterial cell surface. For three monoclonal IgGs considered highly specific for staphylococcal surface glycans, we found that IgM conversion greatly broadened antibody reactivity to both Gram-positive and Gram-negative bacterial pathogens, and even provided *in vivo* cross-protection against *Streptococcus pyogenes*. Altogether, these findings provide an important step change to overcome the narrow specificity hurdle in the development of antibody therapy for bacterial infections.

RESULTS

Conversion of anti-WTA IgG to IgM broadens antibody reactivity

To better understand potential differences between IgG and IgM in bacterial immune defenses, we first focused on *Staphylococcus aureus*, a Gram-positive bacterium that is among the leading causes of hospital- and community acquired infections.[1,27] In the past 10 years, potent IgG1 mAbs against *S. aureus* have been discovered and characterized.[8,28] Several mAbs target the glycopolymer wall teichoic acid (WTA), which is highly abundant on the cell surface and considered as an important target for staphylococcal vaccines and antibody therapies.[29] Interestingly, both on a monoclonal and a polyclonal antibody level, WTA-targeting IgM outperforms IgG in complement activation and opsonophagocytic killing of *S. aureus*.[25,30] Moreover, in patients suffering from *S. aureus* bacteraemia, low levels of WTA-specific IgM, but not IgG or IgA, correlated with disease mortality.[25] For this reason, we aimed to investigate, at a monoclonal level, how conversion of WTA-targeting IgGs into IgM would affect antibody reactivity and functionality.

We first probed anti-WTA antibody 4461, which is derived from an IgG B cell of a *S. aureus*-infected patient. As expected for IgGs, the Fab domains of 4461-IgG have undergone somatic hypermutation *in vivo* to increase antigen specificity.[8] Previous studies with 4461-IgG1 showed that 4461-Fab domains are highly specific for one of two possible anomeric GlcNAc decorations on *S. aureus* WTA[31] (Figure 1A), i.e., α-GlcNAc.[8] We cloned and expressed 4461-Fab domains as IgG1 and as pentameric IgM, which is the predominant form of IgM in human serum.[23] We co-expressed IgM with a J-chain, which is the only circulating IgM oligomer in humans,[23] and validated the presence of pentameric IgM by mass photometry (Figure S1A). Then, we compared the binding of different anti-α-GlcNAc-WTA (4461) IgG and IgM to different *S. aureus* strains. As expected, 4461-IgG and 4461-IgM bound comparably to *S. aureus* LAC, a strain that decorates WTA with a mixture of α- and β-GlcNAc[32] (Figures 1B and S1B). Little to no binding was observed of the 4461-IgG antibodies to Wood46, which lacks α-linked GlcNAc and only expresses β-GlcNAc-WTA[32] (Figures 1C, 1D, and S1C). In contrast, we observed strong binding of 4461-IgM to Wood46, despite the absence of α-linked GlcNAc WTA on this strain (Figures 1C and 1D). IgG binding was not influenced by bacterial immune evasion proteins, since the LAC strain was deleted for IgG-Fc binding proteins SpA and Sbi and the Wood46 strain is naturally low in expression of SpA and Sbi.[32] The cross binding of 4461-IgM to Wood46 was specific for IgM, since no binding was observed for IgG2, IgG3, and IgG4 variants of 4461 (Figure S1C). Moreover, no binding was observed to either *S. aureus* strain with an IgM targeting a non-bacterial antigen trinitrophenol (TNP) (Figures S1B and S1C).

We wondered whether binding of 4461-IgM to Wood46 also induced Fc effector functions. Therefore, we assessed the phagocytosis of *S. aureus* Wood46 by human neutrophils in the presence of human complement. In line with the binding results, 4461-IgM induced effective phagocytosis of *S. aureus* Wood46 in the presence of complement (Figure 1E), which was readily deposited on the surface (Figure S1D). In contrast, 4461-IgG1, anti-TNP IgG1, and anti-TNP IgM did not induce phagocytosis nor complement activation on Wood46 (Figures 1E and S1D). 4461-IgM also induced opsonophagocytic killing of Wood46, whereas 4461-IgG1 could not (Figure S1E). Some reduction in colony forming units (CFUs) was also induced by 4461-IgM without neutrophils present (Figure S1E), likely through agglutination. Thus, the broadened reactivity of 4461-IgM with a *S. aureus* strain lacking α-GlcNAc-WTA translated into complement activation and induction of phagocytosis. To assess whether cross-reactive IgMs can

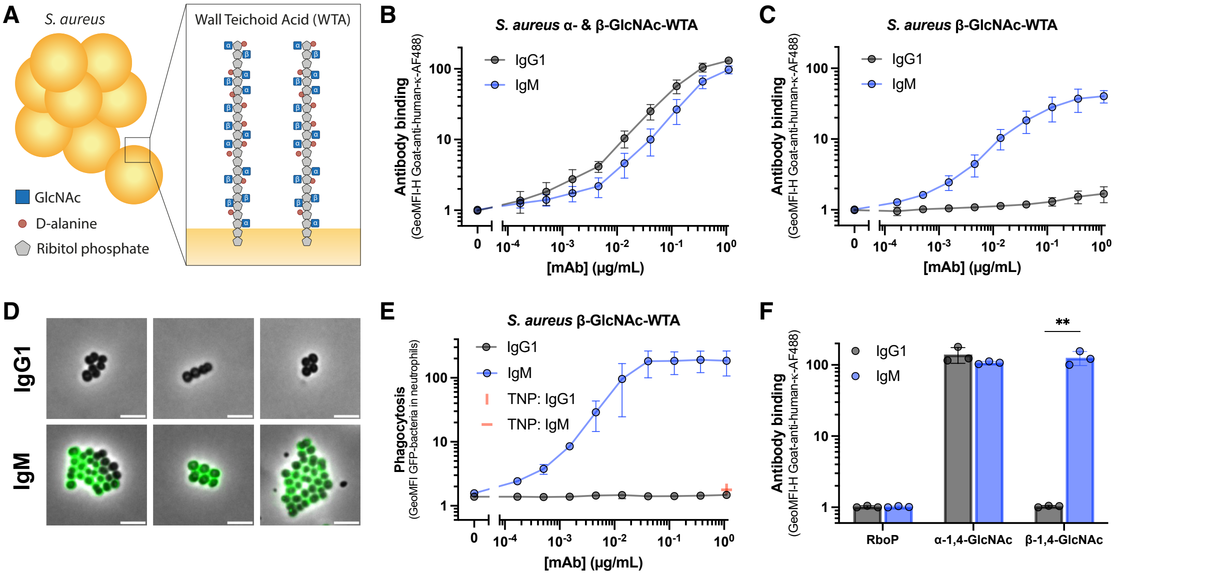

Figure 1. Conversion of anti-WTA IgG1 (4461) to IgM broadens its antigen reactivity with *S. aureus* and WTA

(A–C) (A) Schematic representation of *S. aureus* and WTA structure and glycosylation. Concentration-dependent binding of anti-WTA (4461) IgG1 and IgM to (B) *S. aureus* LAC ΔSpA, sbi::Tn or to (C) *S. aureus* Wood46.

(D) Phase-contrast and widefield fluorescence microscopy images of 1 µg/mL 4461-IgG1 or IgM binding to *S. aureus* Wood46. The white scale bar indicates 5 µm. Images for 4461-IgM have a three times lower exposure time for the GFP channel than the IgG1 images. Microscopy images are representative 20 by 20 µm cutouts of multiple larger images from two independent experiments.

(E) Phagocytosis of GFP-expressing *S. aureus* Wood46 by neutrophils induced by a concentration range of anti-WTA (4461) IgG1 and IgM in 1% ΔIgG/M-serum. As isotype controls, 1 µg/mL anti-TNP IgG1 and IgM were included.

(F) Binding of 1 µg/mL anti-WTA (4461) IgG1 and IgM to synthetic WTA beads with either only the ribitol phosphate (RboP) backbone or treated with TarM or TarS enzymes to add modifications of α-1,4-GlcNAc or β-1,4-GlcNAc respectively. All data represent mean ± SD of three (or four for B) independent experiments. A multiple unpaired *t* test was used to determine significant difference between the indicated samples in f), of which *p* values are indicated with $^{**}p < 0.01$.

activate complement in the presence of pre-existing anti-*S. aureus* antibodies,[25] we spiked 4461-IgM into healthy donor serum and assessed C3b deposition on the bacterial surface. At serum percentages of 1% and higher, we observed C3b-deposition in the absence of spiked 4461-IgM (Figure S1F), likely due to either pre-existing antibodies or the activation of lectin/alternative complement pathways. However, 4461-IgM boosted C3b deposition at lower serum concentrations (0.01%–0.1%, Figure S1F), indicating that cross-reactive IgM can activate complement on *S. aureus* in the presence of pre-existing antibodies.

Clinically relevant *S. aureus* strains express SpA,[33] and we wondered whether this would impact the broadened reactivity of 4461-IgM. Therefore, we selected a *S. aureus* strain, N315, that only expresses β-GlcNAc on its WTA and expresses SpA as confirmed with an anti-SpA antibody (Figure S1G). As expected due to Fcγ-mediated binding to SpA,[34] both 4461-IgG1 and the IgG1 isotype control, but not 4461-IgG3 and its isotype control, bound strongly to N315 (Figure S1H). Similar to our results on Wood46, 4461-IgM, but not IgM isotype, also bound to N315 (Figure S1H). Lastly, we verified that the 4461-IgM binding could induce C3b deposition on N315, while Fc-mediated 4461-IgG1 binding was non-functional (Figure S1I). Therefore, isotype conversion of anti-staphylococcal IgG into IgM does not only broaden the antibody's reactivity but also circumvents IgG evasion molecules.

Structural studies have shown that the binding of 4461-Fab to α-GlcNAc-WTA depends on interactions with both the GlcNAc moiety and the ribitol phosphate (RboP) backbone of WTA.[28,31] We hypothesized that conversion of 4461 to an IgM broadened reactivity to other WTA glycotypes. Therefore, we took WTA out of the bacterial context by assessing the binding to synthetic WTA structures coated to beads containing only a single anomeric GlcNAc modification.[29] As previously published, 4461-IgG1 bound to beads coated with α-GlcNAc-RboP fragments, but not to beads coated with only the RboP backbone or RboP fragments modified with β-GlcNAc (Figure 1F). In contrast, 4461-IgM bound to both α- and β-GlcNAc modified RboP fragments but not to beads containing the RboP backbone (Figure 1F). Thus, 4461-IgG1 exclusively recognizes α-GlcNAc-WTA, whereas conversion of this antibody into IgM broadens the binding to both α- and β-GlcNAc-WTA. To validate these results and check that the isotype conversion did not reduce the antibody's specificity, we used bio-layer interferometry (BLI) to study antibody-antigen docking. BLI confirmed that 4461-IgM binding to α-GlcNAc-WTA was not impaired compared to 4461-IgG1. In addition, 4461-IgM bound to β-GlcNAc-WTA whereas 4461-IgG1 or anti-TNP IgM did not (Figure S2). The observed lower binding of 4461-IgM to β-GlcNAc-WTA compared to α-GlcNAc-WTA (Figure S2) suggests either a lower avidity of 4461-IgM to β-GlcNAc-WTA than α-GlcNAc-WTA, or a discrepancy between WTA display curvature or density on the flat

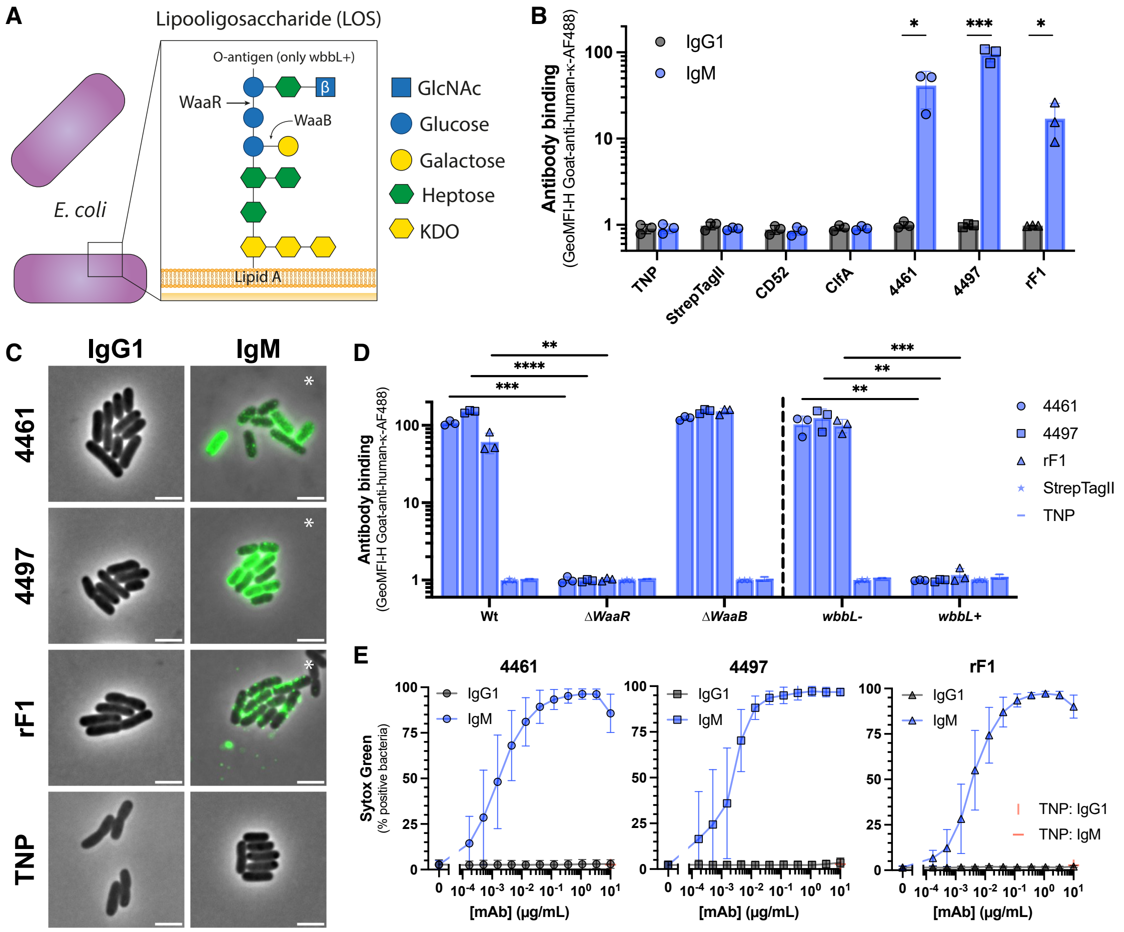

Figure 2. Three anti-staphylococcal IgMs cross-react with *E. coli* and induce complement-dependent lysis

(A–C) (A) Schematic representation of Gram-negative *E. coli* MG1655 lipooligosaccharide (LOS) composition. The inset represents the *E. coli* K-12 LOS structure containing a GlcNAc moiety, depicted as a blue square. The waa mutants with varying sugar structures are indicated with arrows. The O-antigen is not present in any of the strains except wbbL+. Binding of a selection of IgG1 and IgM-converted mAbs to *E. coli* MG1655 (1 μg/mL) as measured by (B) flow cytometry and (C) phase-contrast and widefield fluorescence microscopy. The white scale bar in (C) indicates 5 μm. Image for 4461-, 4497-, and rF1-IgM indicated with an * have a three times lower exposure time for the GFP channel than the other conditions. Microscopy images are a representative 25 by 25 μm cutout of multiple larger images from two independent experiments.

(D) Binding of IgM-converted 4461, 4497, rF1, anti-TNP, and anti-StrepTagII to either *E. coli* BW25113 (Keio) Wt, ΔwaaR, or ΔwaaB, or *E. coli* CGSC7740 wbbL− or wbbL+ (1 μg/mL).

(E) Complement-mediated killing, as measured by Sytox influx, of *E. coli* MG1655 using a concentration range of 4461, 4497, and rF1 IgG1 and IgM (four experiments) or anti-TNP isotype controls (two experiments) in a purified classical pathway assay. All other data represents mean ± SD of three independent experiments. A multiple unpaired *t* test was used to determine significant difference between the indicated bar graphs in which *p* values are indicated with *$p < 0.05$, **$p < 0.01$, ***$p < 0.001$, and ****$p < 0.0001$.

biosensor compared to beads and bacterial cells. Altogether, we show that conversion of a highly specific anti-*S. aureus* IgG into IgM alters the antibody's reactivity toward its antigen.

Converting staphylococcal-specific mAbs from IgG to IgM induces cross-reactivity with *E. coli*

Because 4461-IgM lost its anomeric specificity toward α-GlcNAc-WTA, we wondered whether 4461-IgM binding required the context of the WTA RboP backbone, or whether this antibody could also recognize GlcNAc modifications on other bacterial species. Although Gram-negative bacteria do not produce WTA, GlcNAc is a common monosaccharide in bacterial polysaccharides. For example, *E. coli* types K-12, R2, and R3 incorporate GlcNAc moieties into the core of their lipopolysaccharide (LPS), the lipooligosaccharide (LOS) (Figure 2A).[35] Therefore, we tested binding of 4461-IgG1 and IgM to a K-12 type *E. coli* strain (MG1655). No binding of 4461-IgG1 was detected, whereas 4461-IgM bound strongly to *E. coli* (Figures 2B and S3A), suggesting that this IgM recognized GlcNAc outside the context of WTA. IgMs recognizing other targets (TNP, StrepTagII peptide, human CD52, and *S. aureus* ClfA) did not bind *E. coli* (Figure 2B). Thus, we show that the IgG to IgM conversion of a mAb specific for a Gram-positive bacterium (*S. aureus*) conferred binding to a Gram-negative bacterium (*E. coli*).

CellPress
OPEN ACCESS

We wondered whether our findings with mAb 4461 were clone-specific or could be extended to other antibodies. Therefore, we included two other mAbs targeting glycosylated staphylococcal antigens: anti-WTA (4497), which recognizes β-GlcNAc-WTA[28]; and anti-SDR (rF1), which recognizes O-linked GlcNAc modifications on staphylococcal serine-aspartate dipeptide-repeats proteins.[36] Both mAbs were derived from IgG B cells of *S. aureus*-infected patients and have undergone somatic hypermutation.[28,36] Similar to 4461, structural studies have shown that 4497 interacts with both the GlcNAc moiety and the RboP WTA backbone.[31] We produced 4497 and rF1 as IgG1 and IgM and confirmed that these mAbs still bound *S. aureus* (Figures S3D and S3E). Intriguingly, both IgM variants of 4497 and rF1 potently bound to *E. coli* (Figures 2B, S3B, and S3C), while their IgG1 counterparts did not. Antibody binding of the three anti-staphylococcal IgMs to *E. coli* was validated using microscopy (Figure 2C). Together, this shows that conversion of three different staphylococci recognizing antibodies from IgG to IgM induces cross-reactivity to a completely different species.

To verify that the IgMs bound to the terminal GlcNAc in the *E. coli* K-12 LPS, we examined IgM binding to LPS mutants from the KEIO mutant library and a strain with a reconstituted O-antigen.[37] The *ΔwaaR* (RfaJ) mutant lacks the enzyme responsible for incorporating the final three sugar moieties, including the GlcNAc of the LOS core. This resulted in loss of IgM binding compared to the *E. coli* Keio Wt (Figure 2D). The *ΔwaaB* mutant lacks a D-galactose side chain, but has the GlcNAc still incorporated (Figure 2A)[35,38] and all three IgMs still bound to the *ΔwaaB* mutant (Figure 2D). These results suggest that the GlcNAc in the LPS outer core is the target for the cross-reacting 4461-, 4497-, and rF1-IgM on *E. coli*. The used *E. coli* strains lack the outer repeating saccharide structure on their LPS, the O-antigen (Figure 2A). To investigate how the presence of the O-antigen expression affected the accessibility of the GlcNAc, we used an *E. coli* strain *wbbL*+, in which the IS5-element was removed to restore O-antigen expression.[39] Restoration of O-antigen expression resulted in loss of binding of all three IgMs compared to *E. coli wbbL*− that lacks the O-antigen (Figure 2D), likely as a result of GlcNAc shielding.

Finally, we assessed whether the cross-reactive IgMs could induce complement-mediated killing of *E. coli* through formation of the MAC. Previously, we have shown that antibody-dependent complement-mediated killing of *E. coli* MG1655 can be studied using the DNA dye Sytox in a fully purified complement assay that entirely depends on the antibody to initialise.[40] In line with the binding experiments, 4461-, 4497-, and rF1-IgG1 did not induce complement-mediated killing of *E. coli*, whereas the three IgM counterparts induced efficient complement-mediated killing of *E. coli* MG1655 as measured by Sytox influx (Figure 2E). This shows that the converted IgMs recognize part of their primary epitope in a different context and can subsequently also trigger complement activation to eliminate bacteria.

Cross-reacting IgMs do not react with human cells or serum IgG and depend on antigen density

Since human cells also incorporate GlcNAc moieties in surface molecules and their glycocalyx, we assessed antibody binding to human polymorphonuclear leukocytes (PMNs), erythrocytes, and peripheral blood mononuclear cells (PBMCs). However, none of the IgG1 or IgM-converted anti-staphylococcal antibodies interacted with any of the human cell types (Figure 3A). Aspecific binding of the Fc-tail of IgG1 to Fcγ-receptor I on PMNs was blocked with FLIPr-like.[41] In addition to human cells, human antibodies are also glycosylated with several sugar moieties, including GlcNAc moieties[42]; however, we observed no binding of anti-staphylococcal IgMs to serum IgGs coated on microtiter plates (Figures 3B and S3F). In summary, we show that the cross-reactivity of the three anti-staphylococcal IgMs does not extend to human cells or serum IgG.

We hypothesized that the density of GlcNAc moieties on the target cell could explain the discriminatory binding of anti-staphylococcal IgMs to bacterial but not human cells. To test this hypothesis, we used beads coated with synthetic WTA structures similar to F. Here, the WTA beads were coated with synthetic RboP hexamers, which are enzymatically modified by dedicated recombinant glycosyltransferases (TarS or TarM) resulting in modifications with a range of one to six GlcNAc residues. To reduce the "vertical" GlcNAc density, we used completely defined synthetic RboP hexamers that only have one β-GlcNAc residue at the terminal site mimicking β-GlcNAc-WTA, the originally characterized bacterial antigen target of 4497.[43] Reduction of the vertical antigen density completely abolished 4461-IgM binding, but did not significantly influence 4497-IgM and rF1-IgM binding (Figure 3C). Further reduction of the density on the bead (horizontal density) of these fully defined synthetic WTA antigens by 100-fold, abolished rF1 binding, whereas, as expected, binding of 4497-IgM to its originally characterized target remained (Figure 3D). Similarly, binding of 4497-IgG1 to these beads with a lower horizontal density remained (Figure S3G), suggesting that strong binding to the original antigenic target is density independent. Together these data show that a high antigen density is a driving factor for the observed cross-reactivity of IgMs.

IgM cross-reactivity requires multivalency and can be partly mimicked by IgG engineering

We wondered whether the multivalency of IgM could explain the observed cross-reactivity for *S. aureus* and *E. coli*. First, assembly into pentamers in solution was prevented by introduction of the C575A mutation in the IgM Fc-tail, as verified by mass photometry (Figure S4A).[44] The monomeric IgMs could still bind their original target on *S. aureus*, whereas binding to *E. coli* was drastically reduced (Figures 4A and S5A). In addition, the ability of the IgM monomers to induce complement-mediated killing was significantly reduced compared to IgM pentamers, but not completely absent (Figure 4B). Next, we employed previously described Fc-engineering strategies to generate variants of IgG that can form multimers in solution. First, we generated IgG hexamers (IgG-RGY) by introducing three mutations (E345R/E430G/S440Y) that induce formation of non-covalent IgG hexamers in solution.[45] Second, we engineered the last 18 amino acid residues of the IgM Cμ domain (μ-tail piece) onto IgG1 to generate covalent IgG1 multimers (IgG1-μtp).[46] Using mass photometry, we validated that the IgG1-RGY and IgG1-μtp variants of 4461, 4497, and rF1 formed hexamers and

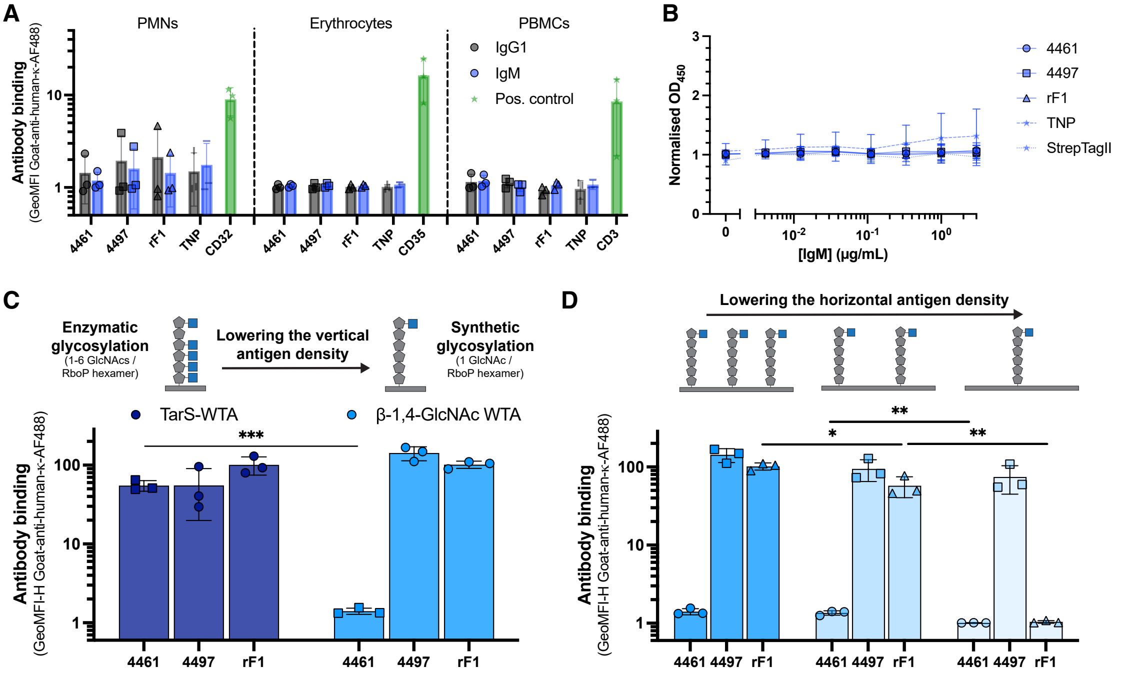

Figure 3. Anti-staphylococcal IgMs do not cross-react with glycans on human cells or serum IgG

(A) Binding of anti-WTA (4461), anti-WTA (4497), anti-SDR (rF1), and isotype control anti-TNP IgG1 and IgM to human PMNs, erythrocytes, and PBMCs (3 μg/mL). A positive control per cell type is indicated with green stars: for PMNs anti-CD32-FITC; for erythrocytes anti-CD35-PE; and for PBMCs anti-CD3-FITC. PMNs were incubated with 10 μg/mL FLIPr-like to block aspecific FcγRI-mediated binding of IgG.

(B) Antibody binding of 4461, 4497, and rF1 IgM and the isotype controls anti-TNP and anti-StrepTagII IgM across a concentration range to ELISA-coated serum IgG.

(C) Binding of anti-WTA (4461), anti-WTA (4497), and anti-SDR (rF1) IgM (1 μg/mL) to enzymatically glycosylated or synthetic RboP hexamers with β-1,4-GlcNAc modifications.

(D) Binding of anti-WTA (4461), anti-WTA (4497), and anti-SDR (rF1) IgM (1 μg/mL) to WTA beads coated with a 10-fold and 100-fold dilution (compared to standard) of synthetic RboP hexamers with one terminal β-1,4-GlcNAc modification.

(C and D) contain a schematic representation of the variation in the vertical and horizontal antigen density on WTA beads. Gray pentagons represent RboP monomers, and blue squares GlcNAc moieties. A multiple unpaired *t* test was used to determine significant differences between antigen densities in which *p* values are indicated with *$p < 0.05$, **$p < 0.01$, and ***$p < 0.001$. Data represents mean ± SD of three independent experiments.

multimers in solution (Figures S4B and S4C) and that they could still bind to *S. aureus* (Figures S5B and S5C) Then we assessed the binding to *E. coli*. Although multimeric variants of 4461-IgG1 and rF1-IgG1 could not cross-react to *E. coli*, we observed strong binding of both 4497-IgG1-RGY and 4497-IgG1-μtp (Figure 4C). Since the LOS of this *E. coli* strain contains β-linked GlcNAc, we speculate that single 4497-Fabs may have a higher affinity for GlcNAc-modified LOS than 4461/rF1-Fabs, and thereby potentially compensate for lower avidity of IgG hexameric platforms (with 6 Fab arms bound[45] compared to IgM with 10 Fab arms bound[47]). Both multimeric variants of 4497-IgG1 could also induce complement-mediated killing of *E. coli* (Figure 4D). When covalent multimerization of 4497-IgG1-μtp was blocked by introduction of the C575S mutation (Figure S4D), binding to and killing of *E. coli* was completely abolished (Figures 4C and 4D). Although it was demonstrated that IgG1-RGY and IgG1-μtp can induce antigen-independent fluid-phase complement consumption *in vitro*[45,48] and *in vivo*,[48] we observed target-specific bacterial killing by 4497-IgG1-RGY and 4497-IgG1-μtp in our *in vitro* model using purified complement proteins. Overall, these data show that the observed cross-reactivity of IgM is conferred by its multivalent structure, which can be partly mimicked through IgG Fc-engineering strategies.

Anti-staphylococcal IgMs cross-react to several bacterial species with exposed GlcNAc

Finally, we wondered whether anti-staphylococcal IgMs could also bind to other bacterial species. Specifically, we selected *Streptococcus pneumoniae* serotype 7F and 9N, *Neisseria meningitidis* serogroup B, and *Streptococcus pyogenes* (commonly referred to as Group A Streptococcus [GAS]), because these bacteria have described surface-exposed GlcNAc moieties and are important human pathogens.[49–51] None of the original IgG1s cross-reacted to these species (Figures 5A and S5D), whereas all three mAbs converted to IgM bound to some of the included bacteria (Figures 5A and S5D). Notably 4461-IgM bound to *S. pneumoniae* and *N. meningitidis*, whereas 4497-IgM and rF1-IgM bound to *S. pyogenes*, suggesting that these converted IgMs retain some level of specificity. Additionally, no binding was observed to a selection of clinical bacterial isolates lacking surface-exposed GlcNAc moieties (Figure S5E). The weak cross-reactivity of 4461 IgM to *N. meningitidis*

CellPress
OPEN ACCESS

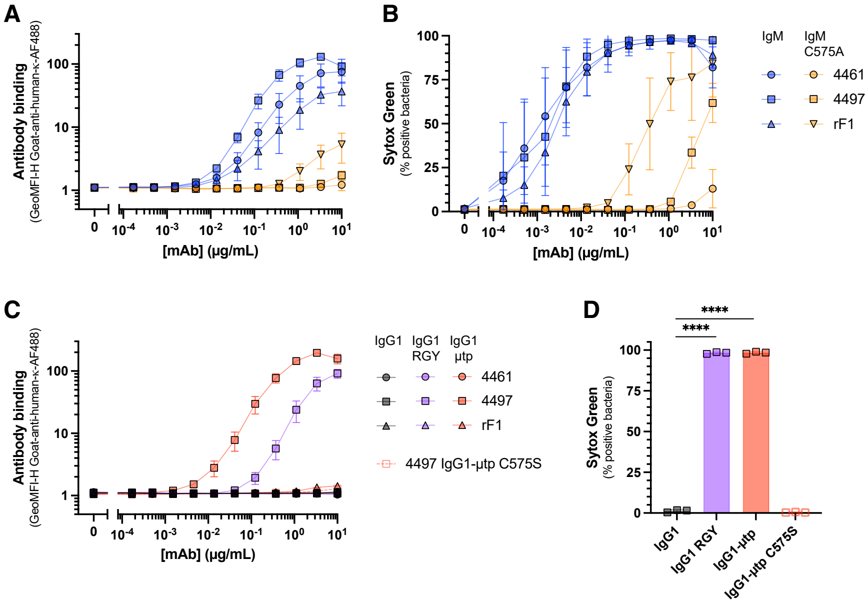

Figure 4. IgM cross-reactivity is due to multivalency

(A and C) Binding of mAbs 4461, 4497, and rF1 across a concentration range and engineered with the following mutations: (A) IgM C575A or (C) IgG1 RGY and IgG1-μtp (C575S) to *E. coli* MG1655.

(B) Sytox positivity of *E. coli* MG1655 after incubation with a concentration range of 4461, 4497, and rF1 IgM C575A in a purified classical pathway assay.

(D) Sytox positivity of *E. coli* MG1655 after incubation with 4497-IgG1, IgG1 RGY, IgG1-μtp, and IgG1-μtp C575S (1 μg/mL) in a purified classical pathway assay. All data represents mean ± SD of three independent experiments. A multiple unpaired *t* test was used to determine significant difference between the indicated bar graphs in which *p* values are indicated with ****$p < 0.0001$.

serogroup B could be explained by the availability of a terminal α-linked GlcNAc in the LOS (Figure S5F).[50] A second β-linked GlcNAc in the LOS is capped with a galactose (Gal) moiety, which requires the LgtB enzyme (Figure S5F).[50] Using the *N. meningitidis* B *ΔlgtB* strain, which no longer produces the terminal Gal unit, we observed binding of 4497-IgM, rF1-IgM, and even 4497-IgG1, although 4497-IgM not significantly (Figure S5G).

To more definitely show the translational prospect of our finding, we studied IgM binding to *S. pyogenes* M1, strain 5448, in more detail. The *S. pyogenes*-specific Group A carbohydrate (GAC) consists of a polyrhamnose backbone modified with (β-1,3-linked) GlcNAc moieties, of which ∼30% is capped with glycerophosphate (Figure 5B).[51] In contrast to IgG mAbs, we observed that the three anti-staphylococcal IgMs could bind to beads coated with fully synthetic GAC modified with GlcNAc, but not without GlcNAc (Figure 5C). These findings confirm that the presence of GlcNAc moieties is required for the observed cross-reactivity of IgM to the *S. pyogenes* GAC. Similar to WTA, high GlcNAc density was important to achieve IgM cross-reactivity to GAC-GlcNAc since lowering both the horizontal and vertical GlcNAc density abolished the binding of 4461-IgM and rF1-IgM, and also lowered the binding of 4497-IgM (Figures S5H and S5I). The reason why 4497-IgM binding was not completely lost on the low-density GAC beads might be due to the fact that the GlcNAc moiety on the GAC beads is β-linked and thus is more similar to β-linked GlcNAc on WTA (4497's original target). Altogether these results demonstrate that conversion of three staphylococcal-specific IgGs to IgM can greatly broaden their reactivity to a wider range of bacterial species with a terminal GlcNAc residue on their surface, but some inherent specificity remains.

Finally, we wondered whether the converted anti-*S. aureus* IgM could also protect against *S. pyogenes* in an *in vivo* bacteraemia model. Mice were passively immunized with 4497-IgM (intravenous [i.v.]) 3 h before infection with *S. pyogenes* (intraperitoneal [i.p.]). We assessed dissemination of *S. pyogenes* from the peritoneal cavity to the liver and spleen 24 h post-infection.[52] Mice treated with 4497-IgM showed significantly lower CFUs counts in the liver in comparison to control groups treated with PBS, 4497-IgG1, or a non-specific IgM antibody (Figure 5D), but not significantly in the spleen (Figure S5J). Together, these data show that anti-*S. aureus* IgM can enhance protection from invasive *S. pyogenes* infection *in vivo*.

DISCUSSION

In this study, we show that *in vitro* isotype conversion of mAbs from IgG to IgM broadens their reactivity toward a range of bacterial species. For three IgG mAbs considered highly specific for staphylococci, we found that modification into IgM alters target specificity and thereby induces cross-reactivity to a range of bacterial species. The converted IgMs retain their capacity to activate downstream effectors functions, e.g., complement activation and bacterial killing *in vitro*, and could protect mice from systemic *S. pyogenes* infection *in vivo*.

Our mechanistic studies indicate that enhanced cross-reactivity by IgM compared to IgG is driven both by changed ligand specificity and multivalent binding. For the WTA-targeting antibodies (4497 and 4461), structural studies have revealed that their ligand specificity depends on multiple interactions between antigen binding domains of the antibody with different residues on the antigen, i.e., that Fab domains interact both with the GlcNAc moiety as well as the RboP backbone.[28,31] In the IgG context, these Fab domains show anomeric specificity to α-GlcNAc or β-GlcNAc WTA glycotypes of *S. aureus*.[25,43] We show that in the context of an IgM pentamer, the same Fab domains can interact with other WTA glycotypes of *S. aureus*, and even recognize other bacterial GlcNAc-modified structures, such as LOS and GAC. The finding that WTA-IgM recognizes GlcNAc moieties outside the context of WTA, strongly suggests that ligand binding via each IgM-Fab domain is reduced to an

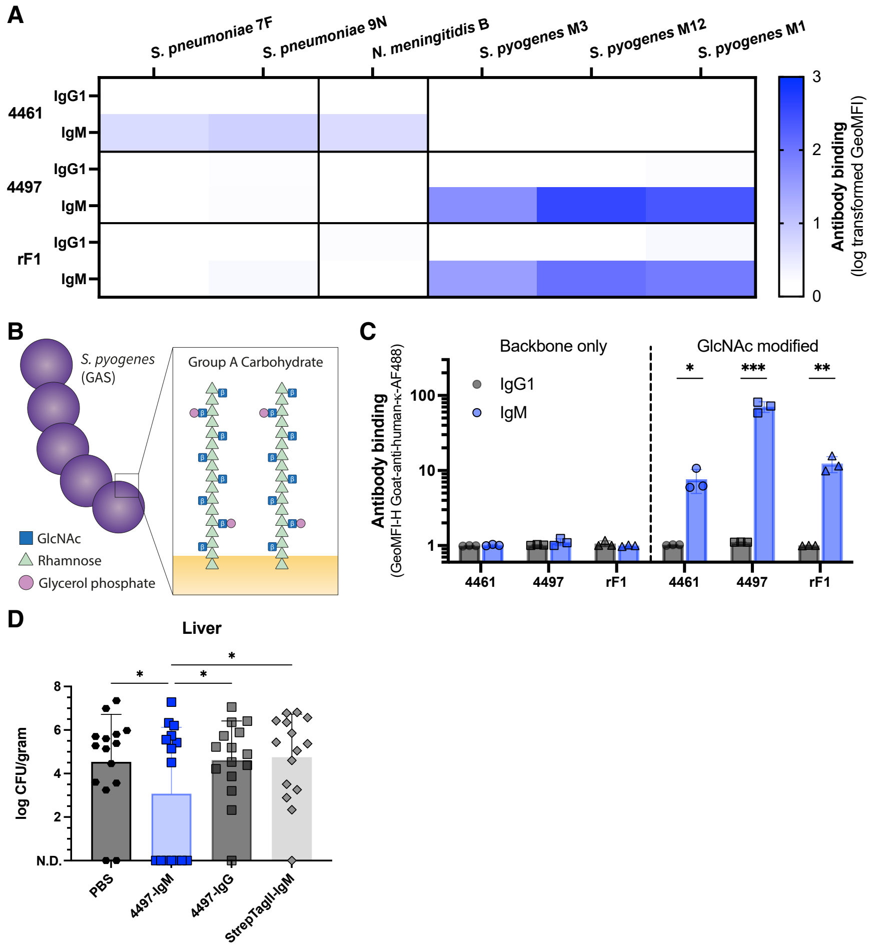

Figure 5. GlcNAc-dependent cross-reactivity of converted anti-staphylococcal IgMs with several human bacterial pathogens and 4497-IgM protection against invasive *S. pyogenes* infection *in vivo*

(A) Heatmap of log transformed binding values of 1 μg/mL anti-WTA (4461), anti-WTA (4497), and anti-SDR (rF1) IgG1 and IgM to a range of bacterial species with described surface-exposed GlcNAc (the bar graph is presented in Figure S5D).

(B) Schematic representation of *S. pyogenes* and the structure and glycosylation of its GAC.

(C) Binding of anti-WTA (4461), anti-WTA (4497), and anti-SDR (rF1) IgG1 and IgM (all 1 μg/mL) to beads coated with synthetic rhamnose hexamers either without (left) or with (right) a β-1,3-GlcNAc modification per rhamnose dimer. Data represents mean ± SD of three independent experiments and a multiple unpaired t test was used to determine significant difference between the indicated bar graphs in which p values are indicated with $^*p < 0.05$, $^{**}p < 0.01$, and $^{***}p < 0.001$.

(D) Colony forming units (CFUs) in liver of mice (n = 15 per group) 24 h post infection with *S. pyogenes* 5448 ($\pm 5 \times 10^7$ CFUs), passively immunized with 50 μg 4497-IgM, 4497-IgG1, anti-StrepTagII-IgM, or PBS. Three independent experiments with five mice per group were performed and pooled for a total of fifteen mice per group. Statistical analysis was performed using a two-way ANOVA comparison ($^*p < 0.05$).

interaction with the GlcNAc moieties (Figure 6). This implies that IgG-Fabs also have a low affinity for non-WTA GlcNAc moieties, but that the binding of a bivalent IgG molecule is too weak to be detected in standard assays. Similarly for the SDR-targeting antibody rF1, we observed that whereas the IgG recognizes GlcNAc moieties on staphylococcal proteins, the IgM variant cross-reacts with other bacteria having only the GlcNAc residues.

Since IgM has ten Fab arms, it can overcome multiple low-affinity interactions due to avidity.[19] In line with this, we show that cross-reactivity is most efficient on surfaces with a high GlcNAc density. In this way, IgM recognition of high-density GlcNAc epitopes can be compared to carbohydrate-recognition molecules (i.e., lectins) of the innate immune system that recognize specific glycan structures on microbial cells.[53,54] For example, mannose-binding lectin (MBL) is an oligomer of multiple polypeptide chains that each recognize monosaccharides such as D-mannose and GlcNAc with low affinity (mM range).[54] However, strong binding (nM range) occurs when sugars are present at a high-density (such as mannan) and several MBL subunits can bind simultaneously.[54] Although eukaryotic cells also express GlcNAc structures on their surface, we observed no binding of WTA-IgMs and SDR-IgM to human cells. A likely explanation is that GlcNAc on human cells is capped with other sugar moieties (e.g. galactose and sialic acid residues[55]) thereby shielded from IgM binding. In addition, in bacterial surface structures, GlcNAc is part of large macromolecular structures (such as GAC, LOS, teichoic acids, and peptidoglycan) in which high-density GlcNAc moieties are part of a highly repeated sugar pattern favoring multivalent binding.[56] Before these IgMs can be employed as therapeutics, we still need to learn more about potential cross-reaction to other self-antigens or commensal bacteria. In conclusion, while staphylococcal targeting IgGs exclusively bind to GlcNAc moieties presented on a particular protein or sugar backbone of staphylococci, their IgM counterparts bind to high-density GlcNAc moieties on other bacterial cells.

The presented findings about IgM's cross-reactivity could help to further understand the role of IgM in bacterial immune defences. Multiple studies have demonstrated that polyclonal IgM, generated in response to vaccination or natural infection and exposure, provides a better correlate of protection against bacterial infection than IgG.[24,25] On a molecular level, it is assumed that IgM is more potent in driving immune-mediated killing of bacteria because of the recruitment of multimeric C1q,[21,47] and/or avoiding interference by bacterial IgG evasion proteins.[25] Our current work raises the question whether *in vivo* produced IgM could also be more cross-reactive than IgG. It should be noted that the IgMs generated in this study are different from earlier described poly-reactive

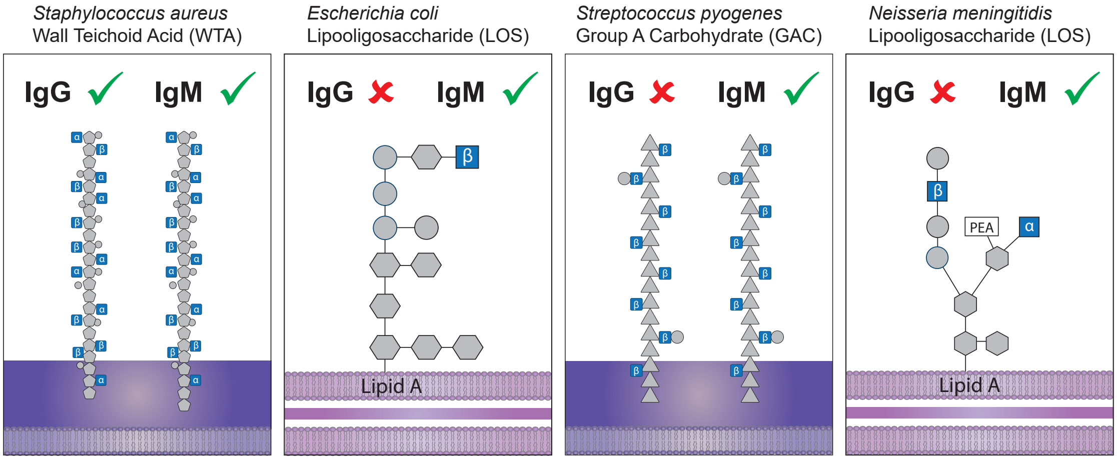

Figure 6. Schematic overview of different bacterial surface structures containing exposed GlcNAc moieties

IgGs targeting *S. aureus* WTA exclusively recognize GlcNAc in a specific anomeric configuration in the context of ribitol phosphate, whereas its IgM counterparts also bind to exposed GlcNAc moieties on other bacterial surface structures, such as LOS from *E. coli*, GAC from *S. pyogenes*, and LOS from *N. meningitidis* serogroup B.

low-affinity IgM (also called "pre-immune" or "natural IgMs").[21,22,57] While pre-immune IgMs have unmutated, germline encoded antigen-binding domains, the *in vitro* converted IgMs presented in this work express affinity-matured Fabs that originate from antigen-experienced, class-switched IgG B cells.[28] *In vivo*, class switching occurs in a unilateral direction: IgM B cells switch to other immunoglobulins but IgGs are never "switched back" to IgM. Therefore, it remains to be determined whether such cross-reactive IgMs with affinity-maturated Fabs are also generated *in vivo*. Potentially, the IgM+ memory B cells found in human circulation that are important for (T cell-independent) immune responses to bacterial infections,[58] are a natural source of broadly reactive glycan-targeting IgMs. An interesting follow-up study would be to study whether vaccines against certain bacterial serotypes also induce IgM-dependent protection against other serotypes (or even other species). A recent study in mice, demonstrated the existence of germline-encoded, pre-immune glycan-specific IgM targeting terminal GlcNAc moieties on a broad range of viral glycoproteins.[59] This, in combination with our work, suggests that the importance of IgM binding to a broader range of species should be considered in research on antibody responses after vaccination or natural infection. Also, further work is needed to better understand the functional differences between IgG and IgM in anti-bacterial immune protection. Although it is well-established that IgM is a more potent inducer of agglutination and MAC formation, the latter is not relevant for immune response against Gram-positive bacteria (such as *S. aureus* and *S. pyogenes*), which largely depends on phagocytosis. Compared to IgG, IgM is unable to bind FcγRs receptors and relies on complement-dependent opsonization to induce phagocytosis.[25] However, unlike IgG, IgM could also interact with Fcα/μ receptors (present on some phagocytic cells and follicular dendritic cells) and Fcμ receptors (present on B and T cells). The antibodies identified in this study could help to further dissect the poorly understood role of Fcμ and Fcα/μ receptors in anti-bacterial immunity.[21]

Our findings warrant investigations into the development of broadly reactive therapeutic antibodies. Monoclonal antibodies are considered as an add-on therapy for infections that cannot be cured by antibiotics alone or as a preventive strategy for patients at risk of developing hospital-acquired infections. However, both in acute and prophylactic settings, the exact strain or even species is often not known at the start of therapy. Therefore, identifying cross-reactive antibodies against multiple bacterial species is an important goal in therapeutic antibody development since these antibodies could be employed similar to empiric antibiotic treatment, i.e., starting treatment in the absence of identification of a definitive causative agent. Although IgM has long been disregarded as a therapeutic agent (because of its poor half-life and large-scale production difficulties), there now is increased interest in the therapeutic use of IgM[21] or engineered IgG molecules that mimic the advantageous properties of IgM.[46,60] Unfortunately, the IgG multimeric platforms used in this study (IgG1-RGY and IgG1-μtp) do not have therapeutic value in their present form because of fluid-phase complement activation in the absence of antigen.[45,48] Future studies are needed to address whether our findings with three anti-staphylococcal IgG mAbs can be extrapolated to other mAbs. Although antibody discovery against bacterial antigens is lagging on other fields, many IgG mAbs against surface antigens are currently being discovered, so it would be interesting to test how IgM conversion affects their reactivity. Based on our mechanistic studies, we expect that the nature of the antigenic target critically determines whether IgG to IgM conversion will induce a broader reactivity. A prerequisite for the cross-reactivity is that a part of the initial epitope (for the mAbs in this study: the GlcNAc moiety) is shared on other bacterial cells. Also, the requirement for a high-density antigen suggests that cross-reaction is more likely for antibodies targeting glycans or glycosylated structures, which are highly

abundant on the bacterial surface.[56] However, cross-reactivity might also be possible for antibodies targeting high-density proteins for which a higher degree of homology is present. In line with this, a previous study identified IgMs that cross-react between a hypha-regulated protein of the fungus *C. albicans* that also bind to structurally related proteins of two Gram-negative bacteria.[61] Next to ligand density, another important aspect affecting IgM binding is the availability of the antigen. Bacterial pathogens are known to shield immunogenic structures using capsule or long repeating O-antigen structures. For example, we showed that restoration of O-antigen expression blocked GlcNAc-targeting IgMs from binding to *E. coli*. In contrast, our results also show that IgMs could cross-react to *S. pyogenes* despite that 30% of the GlcNAc moieties on the GAC are shielded by a negatively charged glycerol phosphate group.[51] Overall, this suggests that cross-reactive IgM antibodies could also be developed against bacterial pathogens with complex surface structures.

In conclusion, our work demonstrates that conversion of antibacterial monoclonal IgG to IgM can broaden their interaction to a range of human-relevant bacterial pathogens. These findings impact our fundamental understanding of IgM in immunity and may accelerate development of broadly reactive antibodies to prevent and treat bacterial infections.

Limitations of the study

In the experimental mouse model, 4497-IgM reduced bacterial burden in the liver but not the spleen. Many mouse infection models fail to accurately mimic human infections in terms of infectious dose and mode of transmission, which could possibly explain the observed differences between organs and the bimodal outcome of our experiments. In addition, the fact that the mice were immunized with human IgM, potentially limits activity or tissue-level distribution in the murine host. Also, while we used an invasive *S. pyogenes* infection model, future studies are needed to validate the therapeutic potential of 4497-IgM in other experimental models of infection, such as those involving the skin. Thus, the exact translational and therapeutic relevance of the here studied IgMs warrants future investigation.

In our *in vitro* studies, we show that cross-reactive IgMs can recognize several human pathogens. It is important to stress that this is only applicable to pathogens with exposed GlcNAc moieties, and we expect no binding to pathogenic strains that shield their GlcNAc moieties with for example an O-antigen or other glycan moieties. Finally, glycan arrays and structural studies could help to pinpoint the exact binding mode of cross-reactive IgMs and further rule out binding to human sugars.

RESOURCE AVAILABILITY

Lead contact

Requests for further information and resources should be directed to and will be fulfilled by the lead contact, Suzan H.M. Rooijakkers (s.h.m.rooijakkers@umcutrecht.nl).

Materials availability

This study did not generate new unique reagents.

Data and code availability

All data reported in this paper will be shared by the lead contact upon request. This paper does not report original code. Any additional information required to reanalyze the data reported in this paper is available from the lead contact upon request.

ACKNOWLEDGMENTS

This project was part of the research program of the Netherlands Centre for One Health (www.ncoh.nl) and received funding from the Netherlands Organisation for Scientific Research (NWO) through Vici Grants (no. 09150182210011 to S.H.M.R; no. 09150181910001 to N.M.v.S.; VI. C.182.020 to J.D.C.C.), the European Research Council (ERC) under the European Union's Horizon 2020 research and innovation program (grant agreement no. 101001937, ERC-ACCENT, to S.H.M.R.), and this work was funded by the Netherlands Organisation for Scientific Research (NWO) through a TTW-NACTAR grant no. 16442 (to S.H.M.R.). This work was supported by a China Scholarship Council (CSC) PhD scholarship (to Z.W. and H.Z.). B.G.J.S. is supported through startup funds from the University of Calgary and Canada Research Chairs program. We would like to thank Mengying Liu, Erik de Vries, and Xander de Haan of the Biomolecular Interaction Facility at Utrecht University for their assistance in setting up and analyzing the BLI experiments.

AUTHOR CONTRIBUTIONS

R.M.M., A.H., D.A.C.H., N.M.v.S., and S.H.M.R. conceived and designed the project. R.M.M. and A.H. conducted most experiments. C.J.C.d.H. and P.C.A. generated monoclonal antibodies. J.W.H. and B.G.J.S. performed/supervised mouse experiments. E.H.T.M.E. and A.J.R.H. performed/supervised mass photometry. Z.W., H.Z., and J.D.C.C. generated/supervised synthetic WTA and GAC oligomers. M.L.B.G. and D.J.D. performed experiments for the revision of the manuscript. R.M.M., D.A.C.H., and S.H.M.R. wrote the manuscript with valuable input from all authors.

DECLARATION OF INTERESTS

N.M.v.S. reports a patent related to vaccine development against *S. pyogenes* (WO 2013/020090 A3, licensee: University of California San Diego, inventors Nina van Sorge and Victor Nizet; licensed by Vaxcyte; personal revenue). S. H.M.R. is listed as coinventor on a patent describing the use of hexamer-enhancing mutations for antibody therapies against *Staphylococcus aureus* (WO2017198731A).

STAR★METHODS

Detailed methods are provided in the online version of this paper and include the following:

- KEY RESOURCES TABLE
- EXPERIMENTAL MODEL AND STUDY PARTICIPANT DETAILS
  - Human material
  - Mouse strains
  - Bacteria strains and culture conditions
- METHOD DETAILS
  - Serum and complement proteins
  - Antibody production
  - Synthetic wall teichoic acid and Group A Carbohydrate beads
  - Antibody binding assay
  - Microscopy
  - Complement activation assay
  - Phagocytosis and opsonophagocytic killing assay
  - Purified classical pathway assay
  - Human cell isolation and antibody binding
  - Serum IgG ELISA
  - Mass photometry
  - Biolayer interferometry
  - Murine model of *S. pyogenes* bacteraemia

CellPress
OPEN ACCESS

- QUANTIFICATION AND STATISTICAL ANALYSIS

SUPPLEMENTAL INFORMATION

Supplemental information can be found online at https://doi.org/10.1016/j.xcrm.2025.102414.

Received: February 21, 2025
Revised: July 28, 2025
Accepted: September 18, 2025
Published: October 13, 2025

REFERENCES

1. GBD 2021 Antimicrobial Resistance Collaborators (2024). Global burden of bacterial antimicrobial resistance 1990-2021: a systematic analysis with forecasts to 2050. Lancet *404*, 1199–1226.
2. Sauvat, L., Verhoeven, P.O., Gagnaire, J., Berthelot, P., Paul, S., Botelho-Nevers, E., and Gagneux-Brunon, A. (2024). Vaccines and monoclonal antibodies to prevent healthcare-associated bacterial infections. Clin. Microbiol. Rev. *37*, e0016022.
3. Motley, M.P., Banerjee, K., and Fries, B.C. (2019). Monoclonal antibody-based therapies for bacterial infections. Curr. Opin. Infect. Dis. *32*, 210–216.
4. Lu, L.L., Suscovich, T.J., Fortune, S.M., and Alter, G. (2018). Beyond binding: antibody effector functions in infectious diseases. Nat. Rev. Immunol. *18*, 46–61.
5. Wardemann, H., and Busse, C.E. (2017). Novel Approaches to Analyze Immunoglobulin Repertoires. Trends Immunol. *38*, 471–482.
6. Edwards, J.C.W., Szczepanski, L., Szechinski, J., Filipowicz-Sosnowska, A., Emery, P., Close, D.R., Stevens, R.M., and Shaw, T. (2004). Efficacy of B-cell-targeted therapy with rituximab in patients with rheumatoid arthritis. N. Engl. J. Med. *350*, 2572–2581.
7. Scott, A.M., Wolchok, J.D., and Old, L.J. (2012). Antibody therapy of cancer. Nat. Rev. Cancer *12*, 278–287.
8. Lehar, S.M., Pillow, T., Xu, M., Staben, L., Kajihara, K.K., Vandlen, R., DePalatis, L., Raab, H., Hazenbos, W.L., Morisaki, J.H., et al. (2015). Novel antibody-antibiotic conjugate eliminates intracellular S. aureus. Nature *527*, 323–328.
9. van der Lans, S.P.A., Bardoel, B.W., Ruyken, M., de Haas, C.J.C., Baijens, S., Muts, R.M., Scheepmaker, L.M., Aerts, P.C., van 't Wout, M.F.L., Preiner, J., et al. (2024). Agnostic B cell selection approach identifies antibodies against K. pneumoniae that synergistically drive complement activation. Nat. Commun. *15*, 8100–8117.
10. Pennini, M.E., De Marco, A., Pelletier, M., Bonnell, J., Cvitkovic, R., Beltramello, M., Cameroni, E., Bianchi, S., Zatta, F., Zhao, W., et al. (2017). Immune stealth-driven O2 serotype prevalence and potential for therapeutic antibodies against multidrug resistant Klebsiella pneumoniae. Nat. Commun. *8*, 1991.
11. Hale, M., Takehara, K.K., Thouvenel, C.D., Moustafa, D.A., Repele, A., Fontana, M.F., Netland, J., McNamara, S., Gibson, R.L., Goldberg, J.B., et al. (2024). Monoclonal antibodies derived from B cells in subjects with cystic fibrosis reduce *Pseudomonas aeruginosa* burden in mice. Preprint at bioRxiv. https://doi.org/10.1101/2024.04.08.588618.
12. Foss, S., Sakya, S.A., Aguinagalde, L., Lustig, M., Shaughnessy, J., Cruz, A.R., Scheepmaker, L., Mathiesen, L., Ruso-Julve, F., Anthi, A.K., et al. (2024). Human IgG Fc-engineering for enhanced plasma half-life, mucosal distribution and killing of cancer cells and bacteria. Nat. Commun. *15*, 2007.
13. Cohen, T.S., Pelletier, M., Cheng, L., Pennini, M.E., Bonnell, J., Cvitkovic, R., Chang, C.S., Xiao, X., Cameroni, E., Corti, D., et al. (2017). Anti-LPS antibodies protect against Klebsiella pneumoniae by empowering neutrophil-mediated clearance without neutralizing TLR4. JCI Insight *2*, e92774.
14. Aguinagalde Salazar, L., den Boer, M.A., Castenmiller, S.M., Zwarthoff, S.A., de Haas, C., Aerts, P.C., Beurskens, F.J., Schuurman, J., Heck, A.J.R., van Kessel, K., and Rooijakkers, S.H.M. (2023). Promoting Fc-Fc interactions between anti-capsular antibodies provides strong immune protection against Streptococcus pneumoniae. eLife *12*, e80669.
15. Gulati, S., Beurskens, F.J., de Kreuk, B.J., Roza, M., Zheng, B., DeOliveira, R.B., Shaughnessy, J., Nowak, N.A., Taylor, R.P., Botto, M., et al. (2019). Complement alone drives efficacy of a chimeric antigonococcal monoclonal antibody. PLoS Biol. *17*, e3000323–e3000329.
16. Ricklin, D., Hajishengallis, G., Yang, K., and Lambris, J.D. (2010). Complement: a key system for immune surveillance and homeostasis. Nat. Immunol. *11*, 785–797.
17. Benn, G., Bortolini, C., Roberts, D.M., Pyne, A.L.B., Holden, S., and Hoogenboom, B.W. (2024). Complement-mediated killing of Escherichia coli by mechanical destabilization of the cell envelope. EMBO J. *43*, 6152–6160.
18. Follador, R., Heinz, E., Wyres, K.L., Ellington, M.J., Kowarik, M., Holt, K.E., and Thomson, N.R. (2016). The diversity of Klebsiella pneumoniae surface polysaccharides. Microb. Genom. *2*, e000073.
19. Sun, Y., Park, M.K., Kim, J., Diamond, B., Solomon, A., and Nahm, M.H. (1999). Repertoire of human antibodies against the polysaccharide capsule of Streptococcus pneumoniae serotype 6B. Infect. Immun. *67*, 1172–1179.
20. Løchen, A., Croucher, N.J., and Anderson, R.M. (2020). Divergent serotype replacement trends and increasing diversity in pneumococcal disease in high income settings reduce the benefit of expanding vaccine valency. Sci. Rep. *10*, 18977.
21. Keyt, B.A., Baliga, R., Sinclair, A.M., Carroll, S.F., and Peterson, M.S. (2020). Structure, Function, and Therapeutic Use of IgM Antibodies. Antibodies *9*, 53.
22. Racine, R., and Winslow, G.M. (2009). IgM in Microbial Infections: Taken for Granted? Immunol. Lett. *125*, 79–85.
23. Oskam, N., den Boer, M.A., Lukassen, M.V., Ooijevaar-de Heer, P., Veth, T.S., van Mierlo, G., Lai, S.H., Derksen, N.I.L., Yin, V., Streutker, M., et al. (2023). CD5L is a canonical component of circulatory IgM. Proc. Natl. Acad. Sci. USA *120*, e2311265120.
24. Simell, B., Nurkka, A., Ekström, N., Givon-Lavi, N., Käyhty, H., and Dagan, R. (2012). Serum IgM Antibodies Contribute to High Levels of Opsonophagocytic Activities in Toddlers Immunized with a Single Dose of the 9-Valent Pneumococcal Conjugate Vaccine. Clin. Vaccine Immunol. *19*, 1618–1623.
25. Hendriks, A., Kerkman, P.F., Varkila, M.R.J., Haitsma Mulier, J.L.G., Ali, S., Ten Doesschate, T., van der Vaart, T.W., de Haas, C.J.C., Aerts, P.C., Cremer, O.L., et al. (2024). Glycan-specific IgM is critical for human immunity to Staphylococcus aureus. Cell Rep. Med. *5*, 101734.
26. Ku, Z., Xie, X., Hinton, P.R., Liu, X., Ye, X., Muruato, A.E., Ng, D.C., Biswas, S., Zou, J., Liu, Y., et al. (2021). Nasal delivery of an IgM offers broad protection from SARS-CoV-2 variants. Nature *595*, 718–723.
27. IHME Pathogen Core Group (2024). Global burden associated with 85 pathogens in 2019: a systematic analysis for the Global Burden of Disease Study 2019. Lancet Infect. Dis. *24*, 868–895.
28. Fong, R., Kajihara, K., Chen, M., Hotzel, I., Mariathasan, S., Hazenbos, W.L.W., and Lupardus, P.J. (2018). Structural investigation of human S. aureus-targeting antibodies that bind wall teichoic acid. mAbs *10*, 979–991.
29. van Dalen, R., Molendijk, M.M., Ali, S., van Kessel, K.P.M., Aerts, P., van Strijp, J.A.G., de Haas, C.J.C., Codée, J., and van Sorge, N.M. (2019). Do not discard Staphylococcus aureus WTA as a vaccine antigen. Nature *572*, E1–E2.
30. Boero, E., Brinkman, I., Juliet, T., van Yperen, E., van Strijp, J.A.G., Rooijakkers, S.H.M., and van Kessel, K.P.M. (2021). Use of Flow Cytometry to Evaluate Phagocytosis of Staphylococcus aureus by Human Neutrophils. Front. Immunol. *12*, 635825.

31. Di Carluccio, C., Soriano-Maldonado, P., Berni, F., de Haas, C.J.C., Temming, A.R., Hendriks, A., Ali, S., Molinaro, A., Silipo, A., van Sorge, N.M., et al. (2022). Antibody Recognition of Different Staphylococcus aureus Wall Teichoic Acid Glycoforms. ACS Cent. Sci. *8*, 1383–1392.
32. Zwarthoff, S.A., Widmer, K., Kuipers, A., Strasser, J., Ruyken, M., Aerts, P. C., de Haas, C.J.C., Ugurlar, D., den Boer, M.A., Vidarsson, G., et al. (2021). C1q binding to surface-bound IgG is stabilized by C1r $_2$ s $_2$ proteases. Proc. Natl. Acad. Sci. USA *118*, e2102787118.
33. Falugi, F., Kim, H.K., Missiakas, D.M., and Schneewind, O. (2013). Role of Protein A in the Evasion of Host Adaptive Immune Responses by Staphylococcus aureus. mBio *4*, e00575.
34. Cruz, A.R., Boer, M.A.d., Strasser, J., Zwarthoff, S.A., Beurskens, F.J., de Haas, C.J.C., Aerts, P.C., Wang, G., de Jong, R.N., Bagnoli, F., et al. (2021). Staphylococcal protein A inhibits complement activation by interfering with IgG hexamer formation. Proc. Natl. Acad. Sci. USA *118*, e2016772118.
35. Heinrichs, D.E., Yethon, J.A., and Whitfield, C. (1998). Molecular basis for structural diversity in the core regions of the lipopolysaccharides of Escherichia coli and Salmonella enterica. Mol. Microbiol. *30*, 221–232.
36. Hazenbos, W.L.W., Kajihara, K.K., Vandlen, R., Morisaki, J.H., Lehar, S. M., Kwakkenbos, M.J., Beaumont, T., Bakker, A.Q., Phung, Q., Swem, L.R., et al. (2013). Novel staphylococcal glycosyltransferases SdgA and SdgB mediate immunogenicity and protection of virulence-associated cell wall proteins. PLoS Pathog. *9*, e1003653.
37. Baba, T., Ara, T., Hasegawa, M., Takai, Y., Okumura, Y., Baba, M., Datsenko, K.A., Tomita, M., Wanner, B.L., and Mori, H. (2006). Construction of Escherichia coli K-12 in-frame, single-gene knockout mutants: the Keio collection. Mol. Syst. Biol. *2*, 2006.0008.
38. Pradel, E., Parker, C.T., and Schnaitman, C.A. (1992). Structures of the rfaB, rfaI, rfaJ, and rfaS genes of Escherichia coli K-12 and their roles in assembly of the lipopolysaccharide core. J. Bacteriol. *174*, 4736–4745.
39. Doorduijn, D.J., Lukassen, M.V., van 't Wout, M.F.L., Franc, V., Ruyken, M., Bardoel, B.W., Heck, A.J.R., and Rooijakkers, S.H.M. (2022). Soluble MAC is primarily released from MAC-resistant bacteria that potently convert complement component C5. eLife *11*, e77503.
40. Muts, R.M., den Boer, M.A., Bardoel, B.W., Aerts, P.C., de Haas, C.J.C., Heck, A.J.R., Rooijakkers, S.H.M., and Heesterbeek, D.A.C. (2023). Artificial surface labelling of Escherichia coli with StrepTagII antigen to study how monoclonal antibodies drive complement-mediated killing. Sci. Rep. *13*, 18836.
41. Stemerding, A.M., Köhl, J., Pandey, M.K., Kuipers, A., Leusen, J.H., Boross, P., Nederend, M., Vidarsson, G., Weersink, A.Y.L., van de Winkel, J. G.J., et al. (2013). Staphylococcus aureus formyl peptide receptor-like 1 inhibitor (FLIPr) and its homologue FLIPr-like are potent FcγR antagonists that inhibit IgG-mediated effector functions. J. Immunol. *191*, 353–362.
42. Nimmerjahn, F., Vidarsson, G., and Cragg, M.S. (2023). Effect of posttranslational modifications and subclass on IgG activity: from immunity to immunotherapy. Nat. Immunol. *24*, 1244–1255.
43. Ali, S., Hendriks, A., van Dalen, R., Bruyning, T., Meeuwenoord, N., Overkleeft, H.S., Filippov, D.V., van der Marel, G.A., van Sorge, N.M., and Codée, J.D.C. (2021). (Automated) Synthesis of Well-defined Staphylococcus Aureus Wall Teichoic Acid Fragments. Chemistry *27*, 10461–10469.
44. Sitia, R., Neuberger, M., Alberini, C., Bet, P., Fra, A., Valetti, C., Williams, G., and Milstein, C. (1990). Developmental regulation of IgM secretion: the role of the carboxy-terminal cysteine. Cell *60*, 781–790.
45. Diebolder, C.A., Beurskens, F.J., de Jong, R.N., Koning, R.I., Strumane, K., Lindorfer, M.A., Voorhorst, M., Ugurlar, D., Rosati, S., Heck, A.J.R., et al. (2014). Complement Is Activated by IgG Hexamers Assembled at the Cell Surface. Science *343*, 1260–1263.
46. Sopp, J.M., Peters, S.J., Rowley, T.F., Oldham, R.J., James, S., Mockridge, I., French, R.R., Turner, A., Beers, S.A., Humphreys, D.P., and Cragg, M.S. (2021). On-target IgG hexamerisation driven by a C-terminal IgM tail-piece fusion variant confers augmented complement activation. Commun. Biol. *4*, 1031.
47. Sharp, T.H., Boyle, A.L., Diebolder, C.A., Kros, A., Koster, A.J., and Gros, P. (2019). Insights into IgM-mediated complement activation based on in situ structures of IgM-C1-C4b. Proc. Natl. Acad. Sci. USA *116*, 11900–11905.
48. Smith, R.I., Coloma, M.J., and Morrison, S.L. (1995). Addition of a mu-tailpiece to IgG results in polymeric antibodies with enhanced effector functions including complement-mediated cytolysis by IgG4. J. Immunol. *154*, 2226–2236.
49. Bentley, S.D., Aanensen, D.M., Mavroidi, A., Saunders, D., Rabbinowitsch, E., Collins, M., Donohoe, K., Harris, D., Murphy, L., Quail, M.A., et al. (2006). Genetic Analysis of the Capsular Biosynthetic Locus from All 90 Pneumococcal Serotypes. PLoS Genet. *2*, e31.
50. Steeghs, L., van Vliet, S.J., Uronen-Hansson, H., van Mourik, A., Engering, A., Sanchez-Hernandez, M., Klein, N., Callard, R., van Putten, J.P.M., van der Ley, P., et al. (2006). Neisseria meningitidis expressing lgtB lipopolysaccharide targets DC-SIGN and modulates dendritic cell function. Cell. Microbiol. *8*, 316–325.
51. Edgar, R.J., van Hensbergen, V.P., Ruda, A., Turner, A.G., Deng, P., Le Breton, Y., El-Sayed, N.M., Belew, A.T., McIver, K.S., McEwan, A.G., et al. (2019). Discovery of glycerol phosphate modification on streptococcal rhamnose polysaccharides. Nat. Chem. Biol. *15*, 463–471.
52. Holmes, C.L., Albin, O.R., Mobley, H.L.T., and Bachman, M.A. (2025). Bloodstream infections: mechanisms of pathogenesis and opportunities for intervention. Nat. Rev. Microbiol. *23*, 210–224. https://doi.org/10.1038/s41579-024-01105-2.
53. van Kooyk, Y., and Rabinovich, G.A. (2008). Protein-glycan interactions in the control of innate and adaptive immune responses. Nat. Immunol. *9*, 593–601.
54. Gjelstrup, L.C., Kaspersen, J.D., Behrens, M.A., Pedersen, J.S., Thiel, S., Kingshott, P., Oliveira, C.L.P., Thielens, N.M., and Vorup-Jensen, T. (2012). The Role of Nanometer-Scaled Ligand Patterns in Polyvalent Binding by Large Mannan-Binding Lectin Oligomers. J. Immunol. *188*, 1292–1306.
55. Trinchera, M., Aronica, A., and Dall'Olio, F. (2017). Selectin Ligands Sialyl-Lewis a and Sialyl-Lewis x in Gastrointestinal Cancers. Biology *6*, 16.
56. Silhavy, T.J., Kahne, D., and Walker, S. (2010). The bacterial cell envelope. Cold Spring Harb. Perspect. Biol. *2*, a000414.
57. Ehrenstein, M.R., and Notley, C.A. (2010). The importance of natural IgM: scavenger, protector and regulator. Nat. Rev. Immunol. *10*, 778–786.
58. Weller, S., Reynaud, C.A., and Weill, J.C. (2005). Vaccination against encapsulated bacteria in humans: paradoxes. Trends Immunol. *26*, 85–89.
59. Yang, Y., Treger, R.S., Hernandez-Bird, J., Lu, P., Mao, T., and Iwasaki, A. (2024). A B cell screen against endogenous retroviruses identifies glycan-reactive IgM that recognizes a broad array of enveloped viruses. Sci. Immunol. *9*, eadd6608.
60. Xiang, Y., Xu, J., McGovern, B.L., Ranzenigo, A., Huang, W., Sang, Z., Shen, J., Diaz-Tapia, R., Pham, N.D., Teunissen, A.J.P., et al. (2024). Adaptive multi-epitope targeting and avidity-enhanced nanobody platform for ultrapotent, durable antiviral therapy. Cell *187*, 6966–6980.e23.
61. Youssef, E.G., Zhang, L., Alkhazraji, S., Gebremariam, T., Singh, S., Yount, N.Y., Yeaman, M.R., Uppuluri, P., and Ibrahim, A.S. (2020). Monoclonal IgM Antibodies Targeting Candida albicans Hyr1 Provide Cross-Kingdom Protection Against Gram-Negative Bacteria. Front. Immunol. *11*, 76.

CellPress
OPEN ACCESS

62. Garred, P., Mollnes, T.E., Lea, T., and Fischer, E. (1988). Characterization of a Monoclonal Antibody MoAb bH6 Reacting with a Neoepitope of Human C3 Expressed on C3b, iC3b, and C3c. Scand. J. Immunol. *27*, 319–327.

63. Zwarthoff, S.A., Magnoni, S., Aerts, P.C., van Kessel, K.P.M., and Rooijakkers, S.H.M. (2021). Method for Depletion of IgG and IgM from Human Serum as Naive Complement Source. Methods Mol. Biol. *2227*, 21–32.

64. den Boer, M.A., Lai, S.H., Xue, X., van Kampen, M.D., Bleijlevens, B., and Heck, A.J.R. (2022). Comparative Analysis of Antibodies and Heavily Glycosylated Macromolecular Immune Complexes by Size-Exclusion Chromatography Multi-Angle Light Scattering, Native Charge Detection Mass Spectrometry, and Mass Photometry. Anal. Chem. *94*, 892–900.

STAR★METHODS

KEY RESOURCES TABLE

REAGENT or RESOURCE	SOURCE	IDENTIFIER
**Antibodies**		
Goat-*anti*-human-kappa-AF488	Southern Biotech	Cat#2060-30; AB_2795725
Goat-*anti*-human-kappa-AF647	Southern Biotech	Cat#2060-31; AB_2795726
Mouse-*anti*-C3b labeled with NHS-Alexa Fluor 488 or 647	Garred et al. 1988[62]	Clone bH6 - in house produced
anti-CD32-FITC	BD Pharmingen	Cat#555448; AB_395841
anti-CD35-PE	BD Pharmingen	Cat#559872; AB_397352
anti-CD3-FITC	BioLegend	Cat#300440; AB_2562046
Goat-*anti*-human-IgM-HRP	Southern Biotech	Cat#2020-05; AB_2795603
Goat-*anti*-human-IgG-HRP	Southern Biotech	Cat#2040-05; AB_2795644
**Bacterial and virus strains**		
S. aureus LAC Δ*spa sbi*::Tn	de Vor et al. 2022[61]	N/A
S. aureus Wood46 cru006	de Vor et al. 2022[61]	N/A
S. aureus Wood46 cru006 GFP	de Vor et al. 2022[61]	N/A
E. coli MG1655	Muts et al. 2023[40]	N/A
E. coli BW25113 Wt	Shigen - Keio collection	https://shigen.nig.ac.jp/ecoli/strain/
E. coli BW25113 Δ*WaaR*	Shigen - Keio collection	https://shigen.nig.ac.jp/ecoli/strain/
E. coli BW25113 Δ*WaaB*	Shigen - Keio collection	https://shigen.nig.ac.jp/ecoli/strain/
E. coli CGSC7740 Wt (*wbbL*-)	Benjamin Sellner, Biozentrum, University of Basel	N/A
E. coli CGSC7740 *wbbL*+	Benjamin Sellner, Biozentrum, University of Basel	N/A
S. pneumoniae 7F	NRLBM	N/A
S. pneumoniae 9N	NRLBM	N/A
N. meningitidis B H44/76	NRLBM	N/A
N. meningitidis B H44/76 Δ*lgtB*	Peter van der Ley, Intravacc	N/A
S. pyogenes M3	Gunnar Lindahl, Lund University	N/A
S. pyogenes M12	NRLBM	N/A
S. pyogenes M1 5448	Kansal et al. 2000[63]	N/A
K. pneumoniae 209S	Janssen et al. 2020 (Ref)	N/A
K. pneumoniae SF001	This study	N/A
K. pneumoniae SF002	This study	N/A
P. aeruginosa 567023.1	This study	N/A
E. cloacae 549052.2	This study	N/A
S. typhimurium 12023	This study	N/A
**Biological samples**		
IgG- and IgM-depleted human serum	Zwarthoff et al. 2021[60]	N/A
Human serum albumin (Albuman)	Prothya Biosolutions	RVG: 103585
Bovine serum albumin	Serva	Cat#11930
**Chemicals, peptides, and recombinant proteins**		
RPMI Medium 1640 (1x)	ThermoFisher/Gibco	Cat#52400-025
POROS™ CaptureSelect™ IgM Affinity Matrix	ThermoScientific	Cat#2812892005
HiTrap Protein G High Performance column	Cytiva	Cat#17040401
C1-complex	Complement Technology	Cat#A098
C1-inhibitor	Complement Technology	Cat#A140
C2	Complement Technology	Cat#A112

(*Continued on next page*)

CellPress
OPEN ACCESS

Continued

REAGENT or RESOURCE	SOURCE	IDENTIFIER
C4	Complement Technology	Cat#A105
C8	Complement Technology	Cat#A125
Superdex 200 Increase 10/300 GL	Cytiva	Cat#8-9909-44
Superose 6 Increase 10/300 GL	Cytiva	Cat#29-0915-96
Dynabeads M-280 Streptavidin	ThermoFisher Scientific	Cat#11205D
Alexa Fluor™ 488 NHS Ester	ThermoFisher Scientific	Cat#A20000
Alexa Fluor™ 647 NHS Ester	ThermoFisher Scientific	Cat#11205D
SYTOX™ Green Nucleic Acid Stain	ThermoFisher Scientific	Cat#S7020
TMB substrate	Sigma Aldrich	
Paraformaldehyde - 10% formaldehyde	Polysciences	Cat#04018
HBSS	Capricorn Scientific	Cat#HBSS-1A
Saponin	Sigma Aldrich	Cat#47036-50G-F
Octet Streptavidin biosensors	Sartorius	Cat#18-5019
**Experimental models: Cell lines**		
EXPI293F cells	ThermoFisher Scientific	Cat#A14527
**Experimental models: Organisms/strains**		
Mus Musculus BALB/c	Charles River	Strain Code 028
**Oligonucleotides**		
5′pcDNA34-XbaI: GACCGATCCAGCCTCCGGACTC<br>3-IgM tail (C575S): GAGATATCAAACTCATTACTAACCGGT<br>AGGGATCGAACCCTTTCAGTAAGAGGTG<br>CCGGCGGTGTCGCTC	ThermoFisher Scientific	N/A
**Recombinant DNA**		
**pcDNA-hG1-μtp_Backbone gBlock:** GACCGATCCAGCCTC<br>CGGACTCTAGAGGATCGAACCCTTgAATTCgATATCTCGAG<br>T**GCTAGC**ACCAAGGGCCCATCGGTCTTCCCCCTGGCACCC<br>TCCTCCAAGAGCACCTCTGGGGGCACAGCGGCCCTGGGC<br>TGCCTGGTCAAGGACTACTTCCCCGAACCGGTGACGGTGT<br>CGTGGAACTCAGGCGCCCTGACCAGCGGCGTGCACACCTT<br>CCCGGCTGTCCTACAGTCCTCAGGACTCTACTCCCTCAGCA<br>GCGTGGTGACCGTGCCCTCCAGCAGCTTGGGCACCCAG<br>ACCTACATCTGCAACGTGAATCACAAGCCCAGCAACAC<br>CAAGGTGGACAAGAAAGTTGAGCCC<br>AAATCTTGTGACAAAACTCACACATGCCCACCGTGCCCAGC<br>ACCTGAACTCCTGGGGGGACCGTCAGTCTTCCTCTTCCC<br>CCCAAAACCCAAGGACACCCTCATGATCTCCCGGACCCCT<br>GAGGTCACATGCGTGGTGGTGGACGTGAGCCACGAAGAC<br>CCTGAGGTCAAGTTCAACTGGTACGTGGACGGCGTGGAG<br>GTGCATAATGCCAAGACAAAGCCGCGGGAGGAGCAGTAC<br>AACAGCACGTACCGTGTGGTCAGCGTCCTCACCGTCCTG<br>CACCAGGACTGGCTGAATGGCAAG<br>GAGTACAAGTGCAAGGTCTCCAACAAAGCCCTCCCAGCCC<br>CCATCGAGAAAACCATCTCCAAAGCCAAAGGGCAGCCCC<br>GAGAACCACAGGTGTACACCCTGCCCCCATCCCGGGAG<br>GAGATGACCAAGAACCAGGTCAGCCTGACCTGCCTGG<br>TCAAAGGCTTCTATCCCAGCGACATCGCCGTGGAGTGGG<br>AGAGCAATGGGCAGCCGGAGAACAACTACAAGACCACG<br>CCTCCCGTGCTGGACTCCGACGGCTCCTTCTTCCTCTACA<br>GCAAGCTCACCGTGGACAAGAGCAGGTGG<br>CAGCAGGGGAACGTCTTCTCATGCTCCGTGATGCATGAGG<br>CTCTGCACAACCACTACACGCAGAAGAGCCTCTCCCTGTC<br>TCCGGGTAAACCCACCCTGTACAACGTGTCCCTCGTGAT<br>GAGCGACACCGCCGGCACCTGTTAC**TGA**AAGGGTTCGA<br>TCCCTACCGGTTAGTAATGAGTTTGATATCTC	Integrated DNA Technologies	N/A

(*Continued on next page*)

Continued

REAGENT or RESOURCE	SOURCE	IDENTIFIER
Software and algorithms		
GraphPad Prism 10	GraphPad Software	https://www.graphpad.com/scientificsoftware/prism/
FlowJo V10	FlowJo	https://www.flowjo.com/

EXPERIMENTAL MODEL AND STUDY PARTICIPANT DETAILS

Human material

Human blood was isolated after informed consent was obtained from all subjects in accordance with the Declaration of Helsinki. Approval for healthy volunteers was obtained from the medical ethics committee of the UMC Utrecht (METC protocol 07–125/C). EDTA-plasma was obtained by centrifugation (10 min, 2000 g at 4°C), aliquoted, and stored at −80°C.

Mouse strains

Mouse experiments were approved by the University of Calgary Animal Care Committee (protocol AC22-0049) and were in accordance with the Canadian Council on Animal Care. 10–12 weeks old female BALB/c mice were purchased from the Charles River and housed in a specific pathogen-free facility under standardized conditions of illumination (12h light/12h dark) and temperature (21°C–22°C). Mice were allowed to acclimate for one week prior to experimentation.

Bacteria strains and culture conditions

A list of the used bacterial strains and the corresponding growth medium is depicted in Table S1. For all experiments except with *N. meningitidis* and *S. pneumoniae*, a single bacterial colony was picked from plate to start overnight cultures while shaking or static (in case of *S. pyogenes*) at 37°C. The next day, bacteria were diluted in fresh medium and grown until mid-log phase (OD_{600} 0.4–0.6) shaking at 37°C. At mid-log phase, bacteria were washed and resuspended to OD_{600} = 1 in RPMI 1640 medium (ThermoFisher/Gibco) containing 0.05% human serum albumin (RPMI-HSA) or PBS with 0.1% bovine serum albumin (PBS-BSA; Serva). *N. meningitidis* was directly grown from plate in PBS-BSA to mid-log and treated similarly as above. *S. pneumoniae* was grown on a fresh plate and diluted in PBS-BSA directly to desired OD before use. To obtain an estimation of the relation between OD_{600} and bacterial count, we prepared of each species for one strain the bacterial solution as described above in duplicate, made a serial dilution, and measured a fixed volume by flow cytometry on a MACSQuant VYB (Miltenyi Biotec) or BD FACSCanto II (BD Biosciences). The values corrected for the dilution factor are presented in Table S1.

METHOD DETAILS

Serum and complement proteins

Human pooled serum depleted from IgG and IgM antibodies (ΔIgG/M-serum) was prepared as described previously.[63] In short, EDTA-treated human pooled serum from healthy donors was passed over a POROS CaptureSelect IgM Affinity Matrix (ThermoScientific) and subsequently over a HiTrap Protein G High Performance column (GE Healthcare) using an ÄKTA Pure system (GE Healthcare). After elution, ions were reconstituted by adding 5 mM $CaCl_2$ and 5 mM $MgCl_2$ and stored at −80°C. The complement proteins C1-complex, C1-inhibitor, C2, C4, and C8 were obtained from Complement Technology. C3 was isolated from human plasma, and C5, C6, C7, and C9 were recombinantly expressed in Expi293F cells.[40]

Antibody production

IgG1-4, pentameric IgM, IgG1 RGY, and IgM C575A monoclonal antibodies (mAbs) were produced as described previously.[14,40] For IgG1s containing a C-terminal tailpiece of IgM (μtp), IgG1s were fused with the 18 amino acid wildtype (PTLYNVSLVMSDTAGTCY) or C575S mutant (PTLYNVSLVMSDTAGTSY) IgM μtp.[46] Therefore, a gBlock was ordered from Integrated DNA Technologies and C575S mutagenesis was performed by PCR. The heavy and light chain variable region (VH and VL, respectively) amino acid sequences for each mAb are depicted in Table S2. In short, the VH and VL sequences were cloned into adapted pcDNA34 vectors (ThermoFisher Scientific) and transfected into EXPI293F cells (ThermoFisher Scientific). For pentameric IgM production, a plasmid coding for the J-chain kindly gifted by Theo Rispens, Sanquin was co-transfected with the VH and VL plasmids. After 5 days of expression, the supernatant was collected and purified using an ÄKTA Pure system (GE Healthcare) with a HiTrap Protein G High Performance column (GE Healthcare) for IgG, or a POROS CaptureSelect IgM Affinity Matrix (ThermoScientific) for IgM. Finally, all IgG mAbs (except IgG1-RGY constructs) and IgM C575A were isolated on a Superdex 200 Increase 10/300 GL and IgM mAbs on a Superose 6 Increase 10/300 GL to ≥95% purity. For all mAbs, a sterile working stock was stored at 4°C and a long-term storage stock at −80°C.

Synthetic wall teichoic acid and Group A Carbohydrate beads

The chemical synthesis of different biotinylated oligomers mimicking *S. aureus* WTA has been described previously,[43] as well as the enzymatic glycosylation of *S. aureus* WTA backbone with recombinant glycosyltransferases TarS and TarM.[29] In short, streptavidin-coated beads (Dynabeads M280, ThermoFisher) were coated with biotinylated oligomers (0.17 mM), chemically defined or enzymatically glycosylated, for 15 min at room temperature, followed by five washes with PBS using a magnetic sample rack and stored at 4°C until use. The synthesis of GAC rhamnose oligomers with and without GlcNAc appendages was previously described by Wang et al. (Z. Wang, Thesis Leiden University "Chemical synthesis of fragments of streptococcal cell wall polysaccharides", Chapter 3, https://scholarlypublications.universiteitleiden.nl/handle/1887/137445). The fragments were equipped with an aminohexanol linker, that was used for biotinylation. The resulting oligomers were immobilized on magnetic Dynabeads as described above.

Antibody binding assay

Bacteria were cultured as described above and incubated at a concentration of OD_{600} = 0.0125 in RPMI-HSA or PBS-BSA, denoted as buffer, with antibody concentrations that are indicated per experiment while shaking for 30 min at 4°C. Bacteria were then washed and resuspended in buffer with 3 μg/mL Goat-*anti*-human-kappa-AF488 (Southern Biotech, 2060-30) or 5 μg/mL Goat-*anti*-human-kappa-AF647 (Southern Biotech, 2060-31) detection antibody while shaking for 30 min at 4°C. After incubation, the bacteria were washed again and resuspended in buffer with 1% paraformaldehyde (PFA), and analyzed by flow cytometry on a MACSQuant VYB (Miltenyi Biotec), MACSQuant X (Miltenyi Biotec), or BD FACSCanto II (BD Biosciences).

To assess antibody binding to synthetic WTA beads, $5*10^6$ beads/mL were incubated with 1 μg/mL antibody in PBS-0.1%BSA-0.05%Tween (PBS-BT) for 30 min at 4°C. Subsequently the beads were washed twice with PBS-BT using a magnetic plate holder, and resuspended in 3 μg/mL Goat-*anti*-human-kappa-AF488 for 30 min at 4°C. Afterward, the beads were washed twice with PBS-BT and resuspended, diluted in PBS-BT, and analyzed with a MACSQuant VYB flow cytometer or BD FACSCanto II (BD Biosciences).

Microscopy

Samples with antibody binding were prepared as described above, but instead of PFA fixation, bacteria were concentrated to OD_{600}~1.5, and dried onto 1% agar pads placed onto a coverslip. Samples were imaged using a Leica SP5 confocal microscope with an HC PL APO 100x/1.40 OIL PH3 objective and a GFP filter cube (470/40 excitation, 525/50 emission) (Leica Microsystems). Images were processed using the Fiji software package for ImageJ.

Complement activation assay

Bacteria were cultured as described above and incubated with antibody concentrations that are indicated per experiment in RPMI-HSA while shaking for 15 min at 4°C. Next, ΔIgG/M-serum was added equivalent to 1% serum and bacterial OD_{600} = 0.01 and incubated while shaking for 30 min at 37°C. Bacteria were washed and resuspended in RPMI-HSA with 3 μg/mL monoclonal mouse anti-C3b (Clone bH6[62]), randomly labeled with NHS-Alexa Fluor 488 or NHS-Alexa Fluor 647 (ThermoFisher Scientific), while shaking for 30 min at 4°C. After incubation, the bacteria were washed with RPMI-HSA, resuspended in RPMI-HSA with 1% PFA, and analyzed by flow cytometry.

Phagocytosis and opsonophagocytic killing assay

PMNs were freshly isolated on the day of the experiment using density gradient centrifugation as described previously.[30] For phagocytosis, GFP-expressing Wood46 were cultured as described above and incubated at a bacterial concentration of OD_{600} = 0.05 with antibody as indicated per experiment in RPMI-HSA while shaking for 15 min at 4°C. Then, ΔIgG/M-serum was added to the equivalent of 1% serum and a bacterial OD_{600} = 0.025 and incubated while shaking for 15 min at 37°C. Lastly, PMNs were added for 15 min at 37°C at an MOI of ~10. After this last incubation step, samples were fixed in cold RPMI-HSA with 1%PFA. Phagocytosis was then measured by flow cytometry.

For opsonophagocytic killing, *S. aureus* Wood46 bacteria were cultured as described above, but washed and resuspended in HBSS (Gibco) with 0.1% HSA instead of RPMI-HSA, which is also used in subsequent steps. Bacteria were incubated at a bacterial concentration of OD_{600} = 0.03 with the indicated concentration of antibody and 1% ΔIgG/M-serum while shaking for 30 min at 37°C. Then, PMNs were added to obtain a bacterial concentration of OD_{600} = 0.0085 at an MOI of ~1, for 90 min while shaking at 37°C with 5% CO_2. Subsequently, the PMNs were lysed by adding 0.3% saponin (Sigma) in miliQ water for 5 min on ice, and the remaining bacteria were serially diluted in PBS and plated on Todd Hewitt Agar plates. After overnight incubation at 37°C, the bacterial colonies were counted.

Purified classical pathway assay

The purified classical pathway was set up as described previously.[40] Purified complement proteins were used in concentrations in physiological ratio as present in serum, denoted as percentage serum equivalent. 100% serum contains: 135 μg/mL C1-complex; 180 μg/mL C1-inhibitor; 20 μg/mL C2; 1250 μg/mL C3; 400 μg/mL C4; 70 μg/mL C5; 64 μg/mL C6; 56 μg/mL C7; 55 μg/mL C8; and 60 μg/mL C9. First, bacteria were cultured as described above and incubated at a concentration of OD_{600} = 0.025 with concentrated antibodies, C1-complex, and 1 μM Sytox Green for 15 min while shaking at 4°C. Subsequently without washing, the remaining

complement components were added to achieve a final concentration of all complement proteins of 1.25% serum equivalent and the indicated antibody concentration while shaking for 45 min at 37°C. After the incubation, the samples were diluted in cold RPMI-HSA and immediately measured by flow cytometry on a MACSQuant VYB.

Human cell isolation and antibody binding

Human PMNs, erythrocytes, and PBMCs were freshly isolated on the day of the experiment using density gradient centrifugation as described previously.[30] The PMNs were mixed with 10 μg/mL recombinant FLIPr-like to block IgG receptors.[41] All cell types were incubated at a concentration of 10^6cell/mL with 1 μg/mL of the indicated antibodies in RPMI-HSA for 30 min at 4°C while shaking. Subsequently, the cells were washed and incubated with 3 μg/mL Goat-*anti*-human-kappa-AF488 or isotype controls: PMNs anti-CD32-FITC (BD Pharmingen, 555448); erythrocytes anti-CD35-PE (BD Pharmingen, 559872); and PBMCs anti-CD3-FITC (BioLegend, 300440) while shaking for 30 min at 4°C. After incubation, the cells were washed with RPMI-HAS, resuspended in RPMI-HSA with 1% PFA, and analyzed by flow cytometry.

Serum IgG ELISA

Maxisorp Nunc (ThermoFisher Scientific) plates were coated overnight at 4°C with 3 μg/mL serum IgG or IgM, which was isolated from serum/plasma as described previously.[40] The wells were washed three times with PBS with 0.05% Tween (PBS-Tw) and subsequently blocked with PBS-Tw with 4% BSA for 1 h at RT. After washing three times with PBS-Tw, a 3-fold dilution series of the monoclonal converted IgMs of interest was added in PBS-Tw with 1% BSA for 1 h at RT. After washing three times, the detection antibodies, either 1/5000 Goat-*anti*-human-IgM-HRP (Southern Biotech, 2020-05) or Goat-*anti*-human-IgG-HRP (Southern Biotech, 2040-05), were added for 1 h at RT. After the incubation and washing three times with PBS-Tw, freshly mixed TMB substrate was added until color change was observed and then the reaction was stopped with 1 N H_2SO_4. 3,3′,5,5′-Tetramethylbenzidine (TMB) substrate mixture consisted of 0.11 M Sodium acetate, 0.16 g/L Ureum peroxide, and 0.1 g/L TMB in milliQ water. After the reaction was stopped, the OD_{450} was measured with an iMark microplate reader (BioRad).

Mass photometry

Mass photometry analysis of mAbs was performed on a Refeyn OneMP mass photometer (Refeyn) similar as described before.[40,64] Microscope coverslips (24 mm × 50 mm; Marienfeld) were prepared by sequential cleaning in sonication baths of isopropanol and then MilliQ water (2x), followed by placement of a CultureWell gasket (Grace Biolabs). About 15 μL of PBS was placed in a well for focusing, after which about 3 μL of diluted sample was mixed in, with measurement concentrations typically around 5–20 nM. Measurements were recorded using medium field-of-view settings for 60 s. An in-house calibration mix consisting of IgG-halfbody (73 kDa), IgG1 (149 kDa), Apoferretin (VitroEase) (513 kDa), and IgM+J (967 kDa) was used. Recordings were processed in DiscoverMP (Refeyn), and further data analysis and plotting were performed in Jupyter Notebook using an in-house Python library.

Biolayer interferometry

Biolayer interferometry of mAbs binding was performed on an Octet RED384 (Sartorius) machine. Sixteen Octet Streptavidin biosensors (Sartorius, 18–5019) were equipped for the run, and after 600 s of baseline in PBS with 0.5 mM $CaCl_2$ and 0.25 mM $MgCl_2$ (PBS+), seven sensors were loaded with 1 μM of biotinylated enzymatically modified (TarM) synthetic M; seven with 1 μM of biotinylated enzymatically modified (TarS) synthetic β-GlcNAc-WTA hexamers; one with 1 μM of biotinylated non-GlcNAc-modified RboP hexamers; and one without any fragment in PBS+ for 1200 s. A baseline was established for 300 s in PBS+. Then, the association of a concentration range from 50 μg/mL antibody down in steps of 1.5 times of 4461-IgG1, or 50 μg/mL antibody to the empty or RboP control sensors, was measured for 2150 s and subsequently dissociation in fresh PBS+ was measured for 3600 s. The sensors were regenerated by dipping them 5 s in Glycine buffer with pH = 2 followed by 5 s neutralization in PBS+ for three times. A new baseline was established for 300 s in PBS+. Then association and dissociation of 4461-IgM was measured in a similar manner as for 4461-IgG1, but then with steps of 3600 s. This was followed by regeneration, a new baseline, and subsequently association and dissociation of TNP-IgM for periods of 1800 s. Lastly, the sensors were regenerated and a last baseline in PBS+ was established for 300 s in PBS+.

Murine model of *S. pyogenes* bacteraemia

Mouse experiments were approved by the University of Calgary Animal Care Committee (protocol AC22-0049) and were in accordance with the Canadian Council on Animal Care. 10–12 weeks old female BALB/c mice were purchased from the Jackson Laboratory and housed in a specific pathogen-free facility under standardized conditions of illumination (12h light/12h dark) and temperature (21°C–22°C). Mice were allowed to acclimate for one week prior to experimentation. *S. pyogenes* 5448 wildtype was grown overnight from a freshly streaked blood agar plate at 37°C + 5% CO_2. Overnight culture was diluted 1:2000 in THB (BD bioscience) + 1% yeast extract (Bioshop) and grown to an OD_{600} of 0.4, followed by two washes with PBS. Mice were passively immunized intravenously (i.v.) with monoclonal antibodies (50 μg in 150 μL PBS) through a tail vein catheter. After 3h, mice were infected with *S. pyogenes* 5448 ($\pm 5*10^7$ CFU) by intraperitoneal (i.p.) injection. After 24 h, mice were anesthetized with inhaled isoflurane (Fresenius Kabi) and blood was collected in 40 μL heparin (20 U/mL, BD biosciences through cardiac puncture). Spleen and liver were harvested, weighed, and homogenized in 1 mL PBS (VWR 200 Homogenizer). Samples were plated in serial dilution (undiluted, 1:10, 1:100,

1:1000) in stripes of 30 μL to form 4 streaks per sample on blood agar plates and incubated overnight at 37°C + 5% CO_2. Bacterial colonies were counted and CFU per gram tissue was calculated.

QUANTIFICATION AND STATISTICAL ANALYSIS

Flow cytometry data were analyzed using FlowJo V10 (FlowJo LLC) and, where indicated, subsequently normalized over the detection antibody controls in Excel (Microsoft). Graphs including standard deviation bars were constructed with Graphpad Prism 10 software, which was also used for statistical analysis indicated per figure where applied. Except for the murine model of bacteraemia, a multiple unpaired t test was used to determine significant difference between the indicated samples in bar graphs, which are indicated with *, **, ***, or **** representing *p*-values of <0.05, 0.01, 0.001, 0.0001, respectively. For the murine model of bacteraemia, the results were statistically analyzed using two-way ANOVA with Turkey's multiple comparisons test. A *p*-value under 0.05 was considered statistically significant.

Cell Reports Medicine, Volume *6*

Supplemental information

Isotype conversion of Staphylococcal-specific IgG into IgM broadens the reactivity to other bacterial pathogens

Remy M. Muts, Astrid Hendriks, Josefien W. Hommes, Max L.B. Grönloh, Douwe J. Dijkstra, Carla J.C. de Haas, Piet C. Aerts, Eduard H.T.M. Ebberink, Albert J.R. Heck, Zhen Wang, Haoru Zhuang, Jeroen D.C. Codée, Bas G.J. Surewaard, Dani A.C. Heesterbeek, Nina M. van Sorge, and Suzan H.M. Rooijakkers

Supplementary Material

Isotype conversion of Staphylococcal-specific IgG into IgM broadens the reactivity to other bacterial pathogens

Remy M. Muts[1], Astrid Hendriks[2], Josefien W. Hommes[3], Max L.B. Grönloh[1], Douwe J. Dijkstra[1], Carla J.C. de Haas[1], Piet C. Aerts[1], Eduard H.T.M. Ebberink[4], Albert J.R. Heck[4,5], Zhen Wang[6], Haoru Zhuang[6], Jeroen D.C. Codée[6], Bas Surewaard[3], Dani A.C. Heesterbeek[1], Nina M. van Sorge[2,7], Suzan H.M. Rooijakkers[1,*]

[1]Department of Medical Microbiology, University Medical Center Utrecht, Utrecht, The Netherlands.
[2]Department of Medical Microbiology and Infection Prevention, Amsterdam UMC, University of Amsterdam, Amsterdam, The Netherlands.
[3]Department of Microbiology, Immunology and Infectious Diseases, Snyder Institute for Chronic Diseases, Cumming School of Medicine, University of Calgary, Calgary, AB T2N 4N1, Canada.
[4]Biomolecular Mass Spectrometry and Proteomics, Bijvoet Center for Biomolecular Research and Utrecht Institute of Pharmaceutical Sciences, Utrecht University, 3584 CH Utrecht, The Netherlands.
[5]Netherlands Proteomic Center, 3584 CH Utrecht, The Netherlands.
[6]Leiden Institute of Chemistry, Leiden University, 2333 CC Leiden, The Netherlands.
[7]Netherlands Reference Laboratory for Bacterial Meningitis, Amsterdam UMC, Amsterdam, The Netherlands.

*Corresponding Author: Suzan Rooijakkers, Email: s.h.m.rooijakkers@umcutrecht.nl

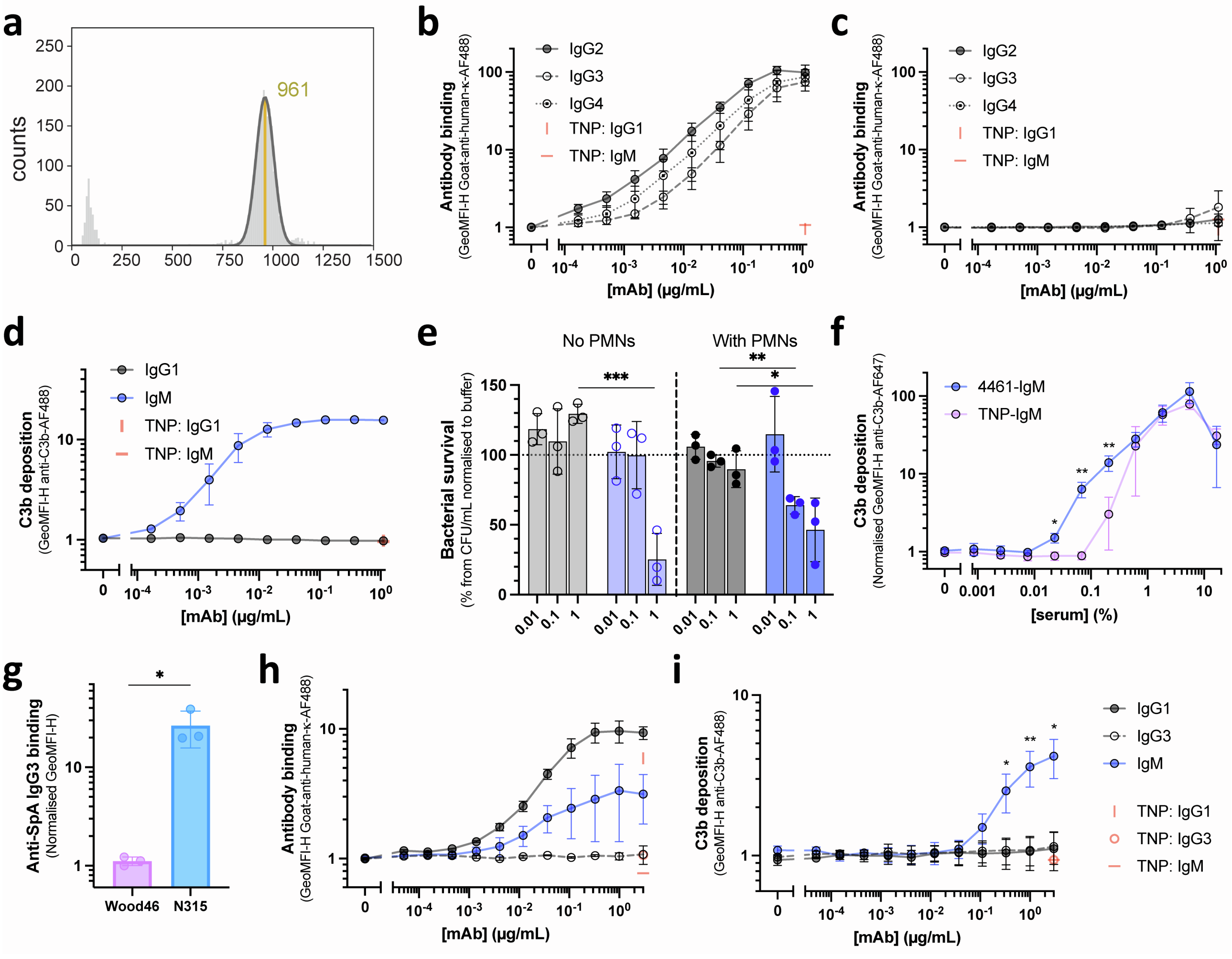

Figure S1. Control experiments to validate the quality, specificity, and functionality of the recombinant IgMs. Related to figure 1.

a) Mass photometry analysis of recombinant pentameric IgM. Mass histograms with Gaussian fits of the peaks of mass photometry analysis of recombinant anti-WTA (4461) IgM co-expressed in the presence of a J-chain. Bandwiths of 10 kDa were used and annotated mass correspond to local maxima in density. A minor peak was observed below 90 kDa, this is most likely not a real binding event, but generated as artefacts of background interactions with the glass plate and are typically observed on the Refeyn MP instrument used here. **b, c) 4461 IgG2-4 binding to *S. aureus* LAC and Wood46.** Concentration-dependent binding of anti-WTA (4461) IgG2, IgG3, and IgG4 and 1 µg/mL isotype controls anti-TNP IgG1 and IgM to b) *S. aureus* LAC Δspa, sbi::Tn or to c) *S. aureus* Wood46. **d, e) 4461 IgG1 and IgM mediated complement activation and OPK.** d) C3b deposition induced by a concentration range of anti-WTA (4461) IgG1 and IgM in 1 % ΔIgG/M-serum on Wood46. As isotype controls, 1 µg/mL anti-TNP IgG1 and IgM were included. e) Normalised CFU/mL values over buffer control of *S. aureus* Wood46 incubated in a opsonophagocytic killing (OPK) killing assay with human neutrophils. Bacteria were first incubated with the in the graph indicated concentration in µg/mL of either 4461-IgG1 (in grey) or 4461-IgM (in blue) with 1% ΔIgG/M-serum or buffer, and subsequently either with or without human PMNs. After incubation the neutrophils were lysed with saponin and remaining bacteria were plated and grown overnight before CFU enumaration. A multiple unpaired t test was used to determine significant differences in which p-values are indicated with * < 0.05, ** < 0.01, or *** < 0.001. **f) 4461-IgM can induce complement activation in the presence of competing antibodies.** C3b deposition induced by 1 µg/mL anti-WTA (4461) IgM or anti-TNP IgM in a concentration range of health donor serum on *S. aureus* Wood46. A multiple unpaired t test was used to determine significant differences between the antibodies at erach concentration in which p-values are indicated with * < 0.05, or ** < 0.01. **g – i) 4461-IgM cross-reactive binding and complement activation occur despite the expression of SpA.** g) Detection of SpA expression with an anti-SpA IgG3 antibody of *S. aureus* Wood46 or N315. Values were normalised over an anti-DNP IgG3 antibody control. A multiple unpaired t test was used to determine significant differences between the strain in which p-value is indicated with * < 0.05. h) Concentration-dependent binding of anti-WTA (4461) IgG1, IgG3, and IgM and anti-TNP isotype controls to *S. aureus* N315. i) C3b deposition induced by a concentration range of anti-WTA (4461) IgG1, IgG3, and IgM and anti-TNP isotype controls 1 % ΔIgG/M-serum on N315. A multiple unpaired t test was used to determine significant differences between IgG1 and IgM at each concentration in which p-values are indicated with * < 0.05, or ** < 0.01. All data represents mean ± SD of three independent experiments.

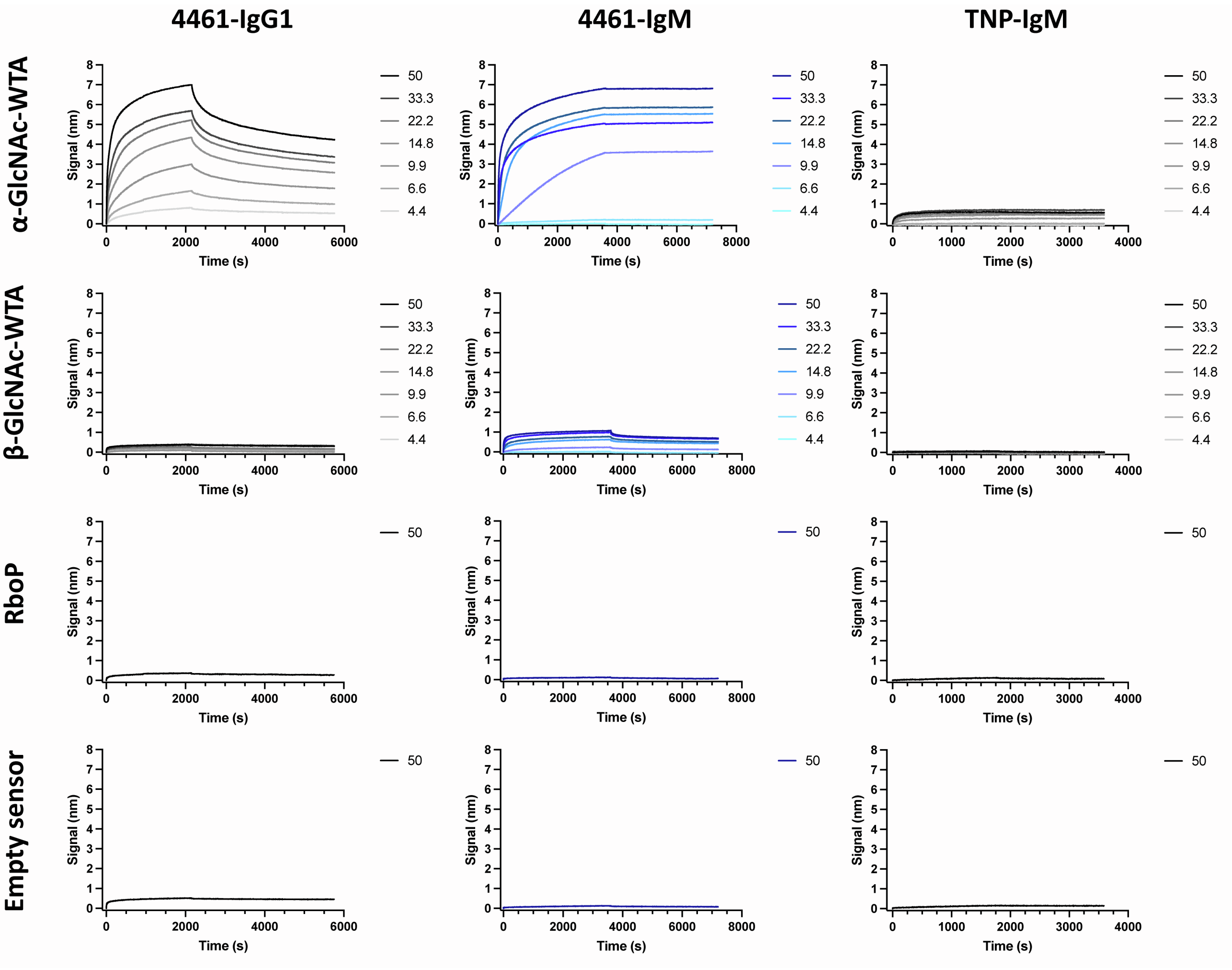

Figure S2. Bio-layer interferometry confirms 4461-IgM cross-reactivity. Related to figure 1.
Bio-layer interferometry measurement of different concentrations of 4461-IgG1, 4461-IgM, and TNP-IgM to streptavidin sensors loaded with either 1 µM biotinylated α-GlcNAc-WTA hexamers, β-GlcNAc-WTA hexamers, RboP hexamers, or nothing. 4461-IgG1 association was measured for 2150 sec, and dissociation for 3600 sec. 4461-IgM association and dissociation were measured for 3600 sec. TNP-IgM association and dissociation were measured for 1800 sec. Legends indicate antibody concentrations in µg/mL.

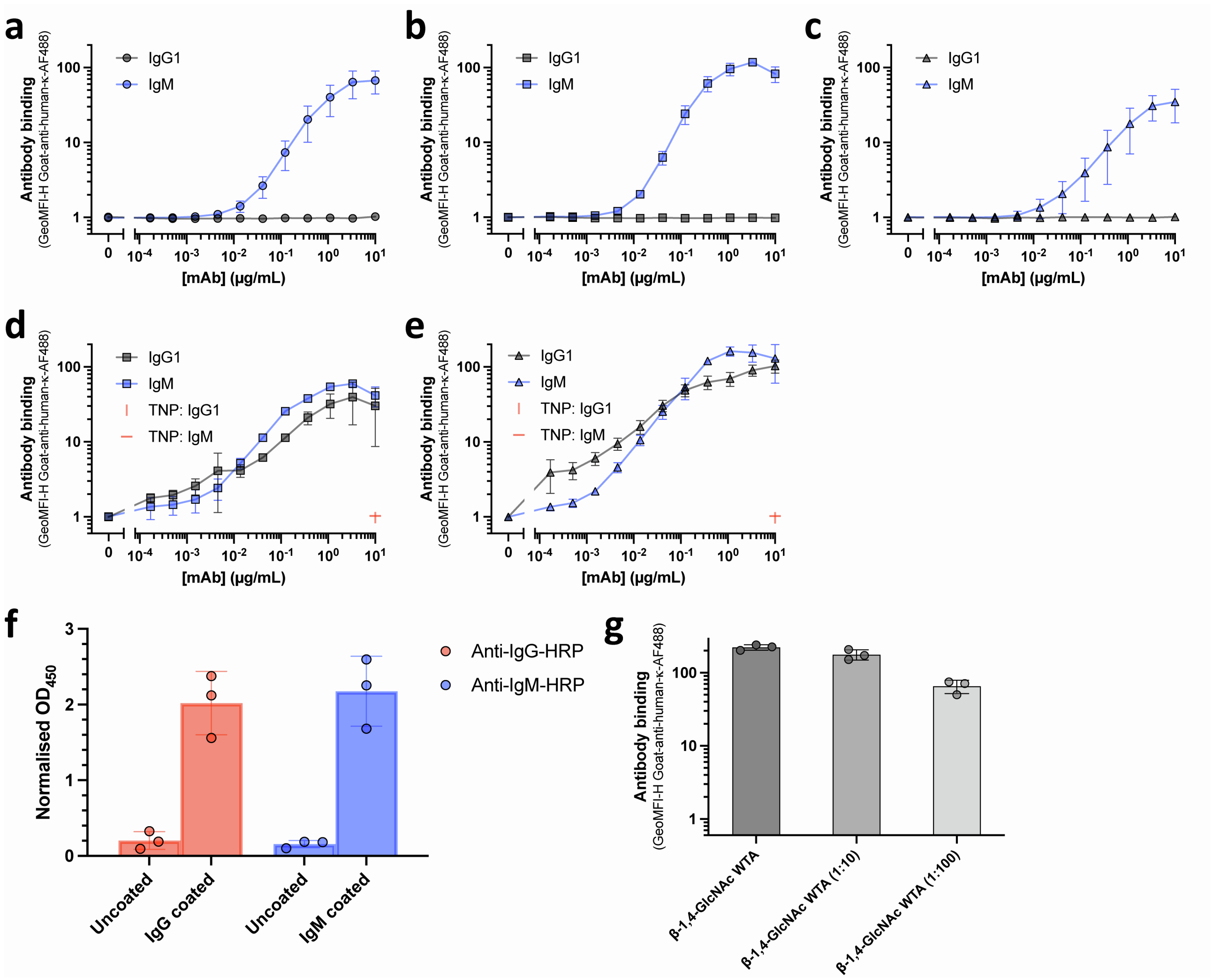

Figure S3. Dose-response curves and controls of antibody binding to *E. coli*, *S. aureus*, and WTA beads. Related to figure 2 and 3.
a – c) 4461, 4997, and rF1 IgG1 and IgM binding curves to *E. coli* MG1655. Concentration-dependent binding of a) anti-WTA (4461), b) anti-WTA (4497), and c) anti-SDR (rF1) IgG1 and IgM to *E. coli* MG1655. **d, e) Validation of 4497 and rF1 IgG1 and IgM binding to *S. aureus*.** Concentration-dependent binding of d) anti-WTA (4497) and e) anti-SDR (rF1) IgG1 and IgM to *S. aureus* LAC Δspa, sbi::Tn. **f) Controls for the serum IgG- and IgM-coated ELISA.** Controls for ELISA with coated serum IgG and IgM. To verify that the coating was successful, the binding of Goat-anti-human-IgG-HRP was assessed to uncoated wells or wells coated with 3 µg/mL serum IgG. As functional control for the anti-IgM detection antibody, its binding was assessed to either uncoated wells, or wells coated with 3 µg/mL serum IgM. **g) 4497-IgG1 binding to horizontaly antigen reduced synthetic WTA beads remains.** Binding of 1 µg/mL anti-WTA (4497) to WTA beads coated with a 10-fold and 100-fold dilution (compared to standard) of synthetic RboP hexamers with one terminal β-1,4-GlcNAc modification. All data represents mean ± SD of three independent experiments.

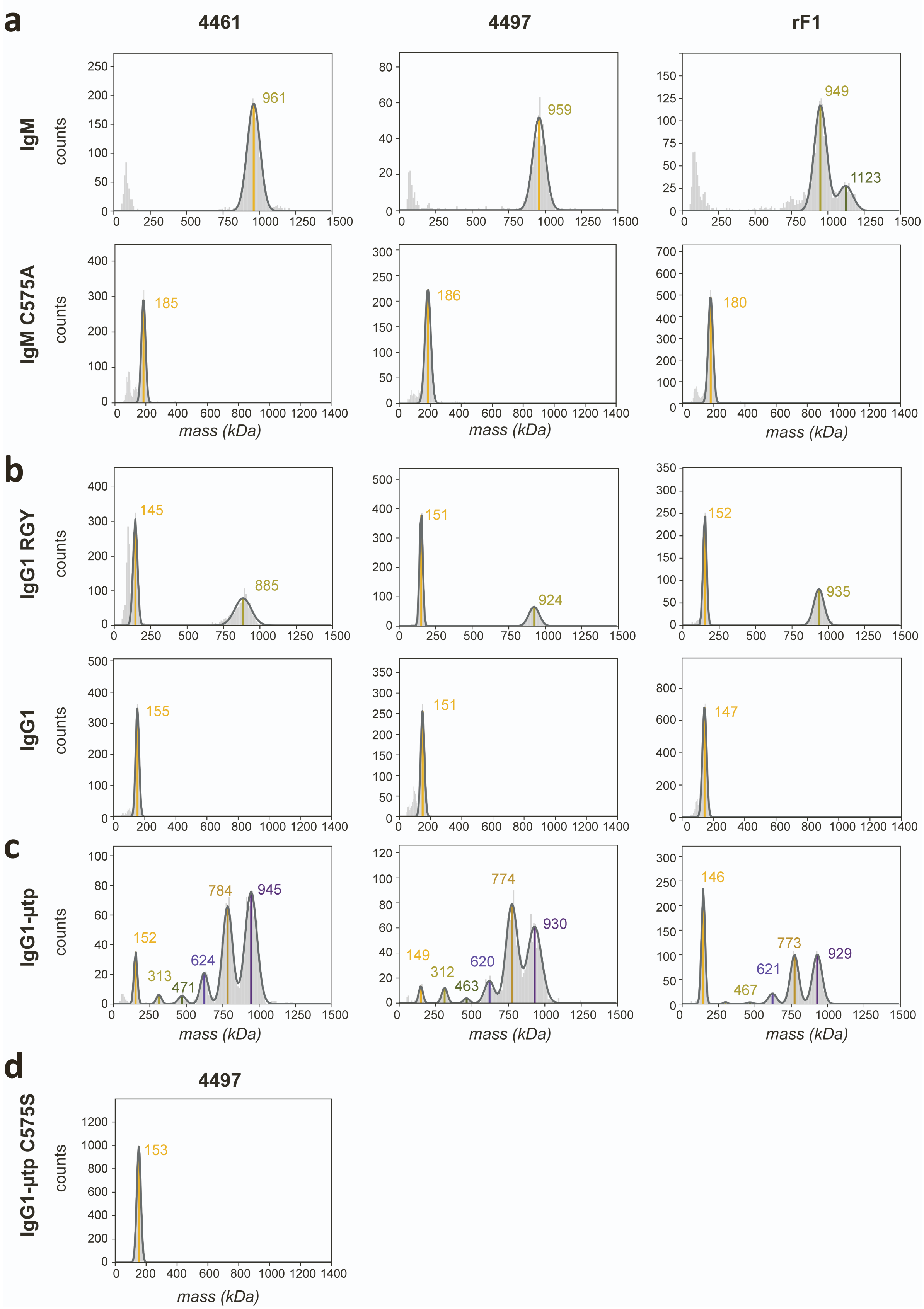

Figure S4. Mass photometry analysis of Ig-constructs to verify oligomerisation status. Related to figure 4. Mass histograms with Gaussian fits of the peaks of mass photometry analysis of recombinant: a) 4461, 4497, and rF1 IgM co-expressed in the presence of a J-chain and IgM C575A monomers; b) IgG1 RGY and IgG1; c) IgG1-µtp; and d) 4497 IgG1-µtp C575S. 4461 IgM is also presented in supplemental figure 1 and is shown here as comparison to 4461 IgM C575A. Bandwiths of 10 kDa were used for IgMs, IgG1-µtps, and IgG1-RGYs or 5 kDA for IgG1s, IgM C575A monomers, and IgG1-µtp C575S. Annotated masses correspond to local maxima in density. In some of the mass histograms minor peaks were observed below 90 kDa. These are most likely not real binding events, but generated as artefacts of background interactions with the glass plate and are typically observed on the Refeyn MP instrument used.

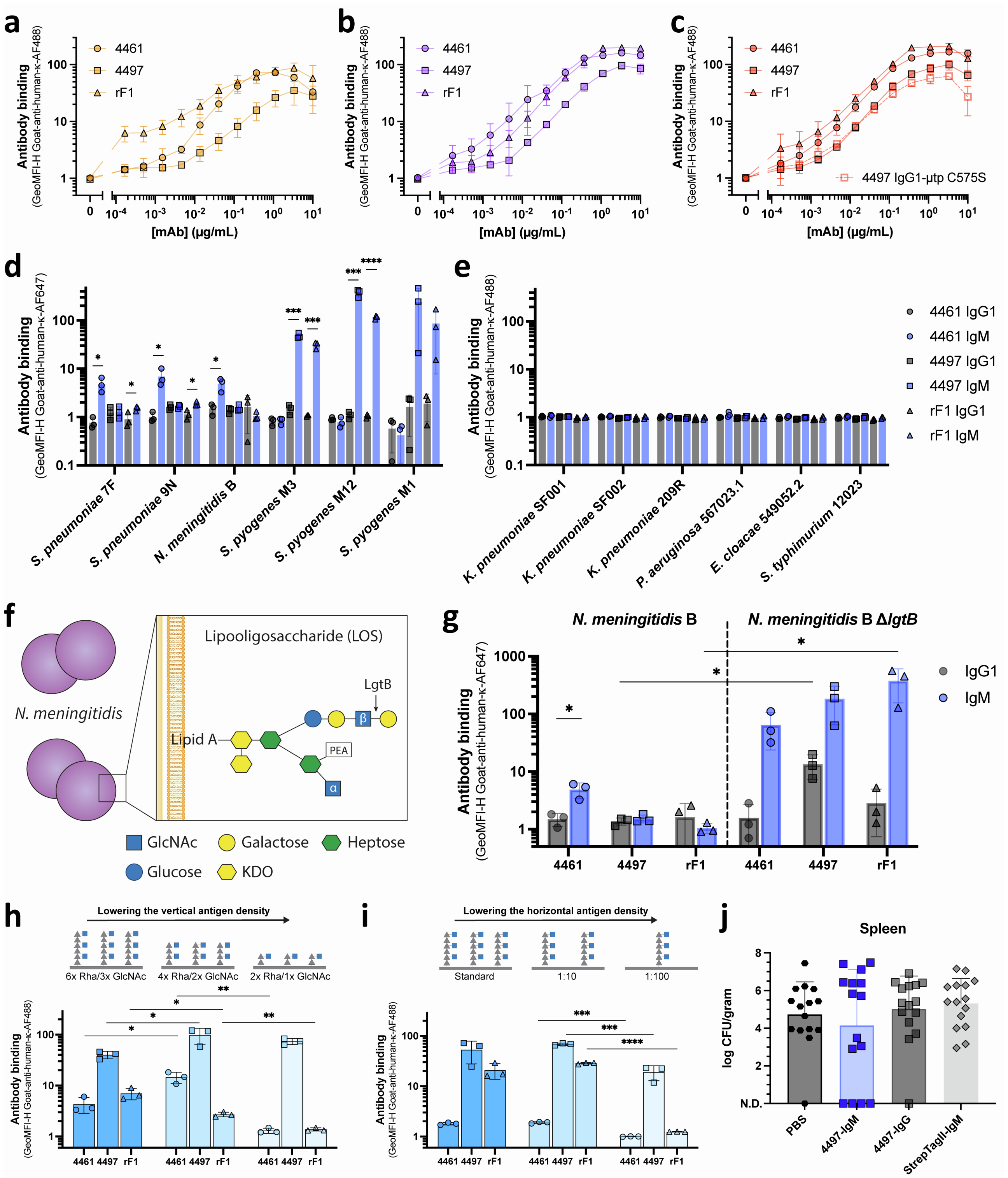

Figure S5. Control experiments related to figure 4 and 5.

a – c) IgG engineered mAbs can bind their original target on *S. aureus*. Binding of 4461, 4497, and rF1 across a concentration range and engineered with the following mutations a) IgM C575A, or b) IgG1 RGY, or c) IgG1-μtp and 4497 IgG1-μtp C575S to *S. aureus* LACΔSpA, sbi::Tn. **d, e) Converted IgMs cross-react to species with surface-expressed GlcNAc.** Binding of 1 μg/mL anti-WTA (4461), anti-WTA (4497), and anti-SDR (rF1) IgG1 and IgM to a range of bacterial species with d) described surface-expressed GlcNAc or e) clinical isolates without described GlcNAc moiety on their surface. A multiple unpaired t test was used to determine significant difference between IgG1 and IgM for each mAb per strain in which p-values are indicated with * < 0.05, ** < 0.01, *** < 0.001 and **** < 0.0001. **f, g) IgM cross-reactivity to *N. meningitidis* is partially obscured by a galactose moiety.** f) Schematic representation of the structure and glycosylation of *N. meningitidis* serogroup B LOS and the incorporation of galactose by LgtB on top of the β-GlcNAc is indicated with an arrow. PEA: phosphoethanolamine. g) Binding of 1 μg/mL 4461, 4497, and rF1 IgG1 and IgM to *N. meningitidis* B Wt (left) and ΔlgtB (right). A multiple unpaired t test was used to determine significant difference between the indicated bar graphs in which p-values are indicated with * < 0.05. **h, i) A high antigen density**

is required for IgM cross-reactivity to GAC beads. h) Binding of anti-WTA (4461), anti-WTA (4497), and anti-SDR (rF1) IgM (1 µg/mL) to GAC beads coated with completely defined synthetic: rhamnose (Rha) hexamers with three β-1,3-GlcNAc modifications, rhamnose tetramers with two GlcNAcs, or rhamnose dimers with one GlcNAc. i) Binding of anti-WTA (4461), anti-WTA (4497), and anti-SDR (rF1) IgM (1 µg/mL) to GAC beads coated with a 10 or 100-fold dilution compared to the standard of completely defined synthetic Rha hexamers with three β-1,3-GlcNAc modifications. h) and i) contain a schematic representation of the variation in the vertical and horizontal antigen density on GAC beads. Gray triangles represent Rha monomers, and blue squares GlcNAc moieties. A multiple unpaired t test was used to determine significant difference between the antigen densities in which p-values are indicated with * < 0.05, ** < 0.01, *** < 0.001 and **** < 0.0001. All data represents mean ± SD of three independent experiments**. j) Spleen CFU of mice infected with *S. pyogenes*.** Colony forming units (CFU) in spleen of mice (n = 15 per group) 24 h post infection with *S. pyogenes* 5448 (±5*10^7 CFU), passively immunised with 50 µg 4497-IgM, 4497-IgG1, anti-StrepTagII-IgM, or PBS.

Table S1. Related to STAR Methods. Bacterial species and strains used.

Bacterial strain	Plate type	Growth medium	Antibiotic	Source	OD_{600} count
S. aureus LAC Δ*spa sbi*::Tn	Blood agar	Todd Hewitt Broth		[61]	
S. aureus Wood46 cru006	Blood agar	Todd Hewitt Broth		[61]	6E8 / mL
S. aureus Wood46 cru006 GFP	Blood agar	Todd Hewitt Broth	10 µg/mL Chloramphenicol	[61]	
E. coli MG1655	LB agar	Lysogeny Broth		[40]	4E8 / mL
E. coli BW25113 Wt	LB agar	Lysogeny Broth		Keio library	
E. coli BW25113 Δ*WaaR*	LB agar	Lysogeny Broth	10 µg/mL Kanamycin	Keio library	
E. coli BW25113 Δ*WaaB*	LB agar	Lysogeny Broth	10 µg/mL Kanamycin	Keio library	
E. coli CGSC7740 Wt (*wbbL*-)	LB agar	Lysogeny Broth		^	
E. coli CGSC7740 *wbbL+*	LB agar	Lysogeny Broth		^	
S. pneumoniae 7F	Blood agar	THY* 5% CO_2		NRLBM**	2E8 / mL
S. pneumoniae 9N	Blood agar	THY* 5% CO_2		NRLBM**	
N. meningitidis B H44/76	Blood agar	Tryptic Soy Broth		NRLBM**	2E8 / mL
N. meningitidis B H44/76 Δ*lgtB*	Blood agar	Tryptic Soy Broth	100 µg/mL Kanamycin	^^	
S. pyogenes M3	Blood agar	THY* 5% CO_2		^^^	
S. pyogenes M12	Blood agar	THY* 5% CO_2		NRLBM**	
S. pyogenes M1 5448	Blood agar	THY* 5% CO_2		[62]	5E7 / mL
K. pneumoniae 209S	LB agar	Lysogeny Broth		UMC Utrecht	3E8 / mL
K. pneumoniae SF001	LB agar	Lysogeny Broth		UMC Utrecht	
K. pneumoniae SF002	LB agar	Lysogeny Broth		UMC Utrecht	
P. aeruginosa 567023.1	LB agar	Lysogeny Broth		UMC Utrecht	1E8 / mL
E. cloacae 549052.2	Blood agar	Tryptic Soy Broth		UMC Utrecht	2E8 / mL
S. typhimurium 12023	LB agar	Tryptic Soy Broth		UMC Utrecht	1E8 / mL

^ Kindly provided by Benjamin Sellner, Biozentrum, University of Basel.
^^ Kindly provided by Peter van der Ley, Intravacc.
^^^ Kindly provided by Gunnar Lindahl, Lund University.
* THY = Todd Hewitt Broth with 0.5% Yeast extract
** Netherlands Reference Laboratory for Bacterial Meningitis

Table S2. Related to STAR Methods. Variable heavy (VH) and variable light (VL) chain region amino acid sequences for each antibody.

Antibody	VH	VL	Source
Anti-WTA (4461)	QVQLVQSGAEVRKPGASVKVSCKASGYSFTDYYMHWVRQAPGQGLEWMGWINPKSGGTNYAQRFQGRVTMTGDTSISAAYMDLASLTSDDTAVYYCVKDCGSGGLRDFWGQGTTVTVSS	DIQMTQSPDSLAVSLGERATINCKSSQSVLSRANNNYYVAWYQHKPGQPPKLLIYWASTREFGVPDRFSGSGSGTDFTLTINSLQAEDVAVYYCQQYYTSRRTFGQGTKVEIK	[8]
Anti-WTA (4497)	EVQLVESGGGLVQPGGSLRLSCSASGFSFNSFWMHWVRQVPGKGLVWISFTNNEGTTTAYADSVRGRFIISRDNAKNTLYLEMNNLRGEDTAVYYCARGDGGLDDWGQGTLVTVSS	DIQLTQSPDSLAVSLGERATINCKSSQSIFRTSRNKNLLNWYQQRPGQPPRLLIHWASTRKSGVPDRFSGSGFGTDFTLTITSLQAEDVAIYYCQQYFSPPYTFGQGTKLEIK	[28]
Anti-SDR (rF1)	EVQLVESGGGLVQPGGSLRLSCAASGFTLSRFAMSWVRQAPGRGLEWVASINSGNNPYYARSVQYRFTVSRDVSQNTVSLQMNNLRAEDSATYFCAKDHPSSGWPTFDSW GPGTLVTVSS	DIQLTQSPSALPASVGDRVSITCRASENVGDWLAWYRQKPGKAPNLLIYKTSILESGVPSRFSGSGSGTEFTLTISSLQPDDFATYYCQHYMRFPYTFGQGTKVEIK	[36]
Anti-StrepTagII	EVQLEQSGPELVKPGASVKMSCKASGYTFTNYYMKWVKQSHGKSLEWIGDLNPNNGDTFYNQKFKGKATLTVDKSSNTAYMQLNSLTSEDSAVYYCARTGRYEENAMDYWGQGTSVTVSS	ELVMTQSPASLAVSLGQRATISCRASESVDSYGKSFMHWYQLKPGQPPKLLIYRASNLESGVPARFSGSGSRTDFTLTIDPVEADDAATYYCQQNNEDPWTFGGGTKLEIK	WO 2015/067768A1
Anti-TNP	EVQIQESGPSLVKPSQTLSLTCSVSGDSITSGYWNWIRKFPGHKIEYMGTISYSGDTYYNPSLKSRISITRDTSKNQYYLHLNSVTTEDTATYYCARYGSYVFDYWGQGTTLTVSS	DVVMTQTPLSLPVSLGDQASISCRSSQSLLHSNGNTYLHWYLQKPGQSPKLLIYKVSNRFSGVPDRFSGSGSGTDFTLKISRVEAEDLGVYFCSQSTHVPWTFGGGTKLEIK	*
Anti-ClfA (Aurex)	QVQLKESGPGLVAPSQSLSITCAISGFSLSRYSVHWVRQPPGKGLEWLGMIWGGGNTDYNSALKSRLSISKDNSKSQVFLKMNSLQTDDTAMYYCARKGEFYYGYDGFVYWGQGTLVTVSA	NIMMTQSPSSLAVSAGEKVTMSCKSSQSVLYSSNQKNYLAWYQQKPGQSPKLLIYWASTRESGVPDRFTGSGSGTDFTLTISSVQAEDLAVYYCHQYLSSYTFGGGTKLEIK	WO2002/WO02072600A2
Anti-CD52 (Campath)	QVQLQESGPGLVRPSQTLSLTCTVSGFTFTDFYMNWVRQPPGRGLEWIGFIRDKAKGYTTEYNPSVKGRVTMLVDTSKNQFSLRLSSVTAADTAVYYCAREGHTAAPFDYWGQGSLVTVSS	DIQMTQSPSSLSASVGDRVTITCKASQNIDKYLNWYQQKPGKAPKLLIYNTNNLQTGVPSRFSGSGSGTDFTFTISSLQPEDIATYYCLQHISRPRTFGQGTKVEIK	WO 2013/181568A2
Anti-SpA (10919)	EVQLVQSGAEVKKPGASVKVSCKASGYTFTSYYMHWVRQAPGQGLEWMGIINPRVGSTSYAQKFQGRVTMTRDTSTSTVYMELSSLRSEDTAVYYCARGRPLSGTGGHHYFDYWGQGTLVTVSS	EIVLTQSPATLSVSPGERATLSCQASQDISNYLNWYQQKPGQAPRLLIYDASNLETGIPARFSGSGSGTEFTLTISSLQSEDFAVYYCQQVYALPPWTFGGGTKVEIK	US 2018/0105584A1

*Kindly provided by Gestur Vidarsson, Sanquin.
